# Different Secondary Metabolite Profiles of Phylogenetically almost Identical *Streptomyces griseus* Strains Originating from Geographically Remote Locations

**DOI:** 10.3390/microorganisms7060166

**Published:** 2019-06-06

**Authors:** Ignacio Sottorff, Jutta Wiese, Matthias Lipfert, Nils Preußke, Frank D. Sönnichsen, Johannes F. Imhoff

**Affiliations:** 1 GEOMAR Helmholtz Centre for Ocean Research Kiel, Marine Microbiology, 24105 Kiel, Germany; isottorff@geomar.de (I.S.); jwiese@geomar.de (J.W.); 2 Facultad de Ciencias Naturales y Oceanográficas, Universidad de Concepción, 4070386 Concepción, Chile; 3 Otto Diels Institute for Organic Chemistry, University of Kiel, 24118 Kiel, Germany; mlipfert@oc.uni-kiel.de (M.L.); npreusske@oc.uni-kiel.de (N.P.); fsoennichsen@oc.uni-kiel.de (F.D.S.)

**Keywords:** *Streptomyces*, geographical isolation, Easter Island, secondary metabolites, 16S rRNA, morphology, High Resolution Mass Spectroscopy (HRMS), Nuclear Magnetic Resonance (NMR), horizontal gene transfer

## Abstract

As *Streptomyces* have shown an outstanding capacity for drug production, different campaigns in geographically distant locations currently aim to isolate new antibiotic producers. However, many of these newly isolated *Streptomyces* strains are classified as identical to already described species. Nevertheless, as discrepancies in terms of secondary metabolites and morphology are possible, we compared two *Streptomyces* strains with identical 16S rRNA gene sequences but geographically distant origins. Chosen were an Easter Island *Streptomyces* isolate (*Streptomyces* sp. SN25_8.1) and the next related type strain, which is *Streptomyces griseus* subsp. griseus DSM 40236^T^ isolated from Russian garden soil. Compared traits included phylogenetic relatedness based on 16S rRNA gene sequences, macro and microscopic morphology, antibiotic activity and secondary metabolite profiles. Both *Streptomyces* strains shared several common features, such as morphology and core secondary metabolite production. They revealed differences in pigmentation and in the production of accessory secondary metabolites which appear to be strain-specific. In conclusion, despite identical 16S rRNA classification *Streptomyces* strains can present different secondary metabolite profiles and may well be valuable for consideration in processes for drug discovery.

## 1. Introduction

The increasing number of pathogens that show antibiotic resistance has triggered the bioprospection of new antibiotics [1]. *Actinobacteria* have shown to be an exceptional source of new antibiotics and pharmaceuticals in general [2]. Within the *Actinobacteria* phylum, *Streptomyces* is the most prolific drug producing genus [3]. *Streptomyces* species have shown an outstanding capacity for the production of secondary metabolites, many of which effectively can treat human diseases. Secondary metabolites of *Streptomyces* species belong to different classes of compounds, such as: polyketides [4], peptides [5] and polyketide-peptides hybrids [6], and have been characterized with different biological activities, such as: antibacterial [7] antifungal [8], anticancer [9] and immune suppression [10].

Biogeographically, *Streptomyces* species have a wide distribution, since they can be found in the most diverse habitats, like polar territories [11], deserts [12], highlands [13], insects [14], marine invertebrates [15] and marine sediments [16,17].

As a result of the current drug bioprospection, multiple *Streptomyces* strains are isolated every year, however only a limited percentage of these bacteria represent new species [18]. Most of them represent already characterized species displaying similar or even identical phylogenetic features [19]. The most widely used method of bacterial characterization is the analysis of the 16S rRNA gene sequence which despite its utility and quickness, has shown ambiguity when discriminating closely related *Streptomyces* strains (>99% of similarity) [20]. Often known *Streptomyces* species are considered to be of little significance for drug discovery due to their lack of novelty in terms of phylogeny and physiology. In consequence, drug discovery may overlook new metabolites produced from *Streptomyces* strains which are closely related to already known strains since they are assumed to be producers of identical secondary metabolites [21]. However, investigations have shown that in addition to the core secondary metabolites, many *Streptomyces* strains have an accessory chemical arsenal which has not been completely studied [22]. Moreover, diverse experiments have demonstrated that changing culture conditions of *Streptomyces* strains may activate cryptic biosynthetic pathways, producing uncommon or unknown strain-specific metabolites [23,24].

The advancement in genome sequencing and the reduction of its cost has allowed the differentiation of closely related *Streptomyces* species at the genomic level [25] and has shed light about the unexploited cryptic biosynthetic pathways harbored by *Streptomyces* strains, or bacteria in general [26]. Despite the genomic advancement, we still lack the understanding of expression and regulation of biosynthetic pathways [26]. This makes it necessary to perform an in vitro characterization of *Streptomyces* strains to obtain specific information of their secondary metabolite features.

To date, few efforts have been made to thoroughly evaluate the differences in secondary metabolite production between two *Streptomyces* strains that are identical according to 16S rRNA phylogeny but originate from different geographic regions [19].

Therefore, we compared two phylogenetically identical *Streptomyces* strains, an isolate from a marine sediment sample from Easter Island, a remote location in the middle of the South Pacific Ocean, and as counterpart a reference strain isolated from Russian garden-soil. Our data showed that the phylogenetically almost identical *Streptomyces* strains shared a number of morphological and chemical features as widely recognized and also expected. However, we also found striking differences in the accessory metabolites produced, which appear to be strain specific. We suggest that these chemical differences may have risen through niche specialization, as well as horizontal gene transfer.

## 2. Materials and Methods

### 2.1. Streptomyces Strains

*Streptomyces* sp. SN25_8.1 was obtained from a marine sediment sample which was collected from the coastal zone of the Easter Island, Chile (27°08′45.0″ S, 109°25′49.8″ W), by the first author (Chilean citizen), in March 2016. The sampling site was outside of the Isla de Pascua national park, and the sample was taken in agreement with regulations by the Chilean government. *Streptomyces griseus* subsp. griseus DSM 40236^T^ was obtained from the German Collection of Microorganisms and Cell Cultures GmbH (DSMZ). This strain was isolated by Krainsky from a garden soil sample from Russia [27,28].

### 2.2. Culture Conditions

Cultivation and morphological comparison of the of *Streptomyces* strains were made using solid Glucose-Yeast extract-Malt extract medium (GYM), 4 g glucose × H_2_O, 4 g yeast extract, 10 g malt extract, 2 g CaCO_3_, 15 g agar, 1 L deionized water, and pH 7.2 [29].

For the evaluation of the secondary metabolites production, the *Streptomyces* strains were grown in Ultra Yield® flasks 2.5 L (Thomson, Oceanside, CA, USA), which contained 1 L of slightly modified Starch-Glucose-Glycerol (SGG) liquid medium [30]. The composition of the production medium was: 5 g soluble starch, 5 g glucose x H_2_O, 5 g glycerol, 1.25 g cornsteep powder, 2.5 g peptone from soymeal, 1 g yeast extract, 1.5 CaCO_3_, and 1 L deionized water. The medium was also supplemented with 15 g/L Tropic Marin™ salt (Wartenberg, Germany). The pH was adjusted to 7.7 using 1 M of HCl and NaOH. The culture was kept in orbital agitation at 240 rpm, 28 °C, for 14 days in darkness.

### 2.3. Molecular Characterization and Phylogenetic Analysis

DNA was extracted from the bacterial cells through the use of DNA isolation kit, DNeasy™ (Qiagen, Hilden, Germany), following the manufacturer instructions. Subsequently, the amplification of the 16S rRNA gene sequence was performed with PCR and the use of general bacterial primers in a concentration of 10 pmol/µL, i.e. 27f, 1492r [31,32], 1387r [33] and 1525r [34].

PCR reagents were obtained from GE Healthcare illustra™ PuReTaq Ready-To-Go™ PCR Beads (GE Healthcare, Glattbrugg, Switzerland) containing DNA polymerase, MgCl_2_, and dNTPs. The PCR conditions were the same as reported by Staufenberger et al. [32]. The 16S rRNA genes were sequenced at the Center for Molecular Biosciences (ZMB) at Kiel University using the primers 27f [31], 342f [32], 534r [32] and 1525r [34]. The 16S rRNA gene sequences were manually curated using Chromas pro software, version 1.7.6 (Technelysium Pty Ltd, Tewantin QLD, Australia) and saved in FASTA format. Primary phylogenetic characterization of the *Streptomyces* strains was achieved using nucleotide NCBI-BLAST and EZbioCloud [35]. Subsequently, the obtained sequences were standardized according to the global SILVA alignment for rRNA genes [36]. This primary alignment was visually compared using ExPASy (SIB bioinformatics resource portal) [37] to determine the level or similarity or divergence of both *Streptomyces* 16S rRNA gene sequences. For the construction of a *Streptomyces* phylogenetic tree, we retrieved the next related type strains 16S rRNA gene sequences from NCBI. All the 16S rRNA gene sequences were gathered in a single FASTA file and aligned in SINA-SILVA web platform [36]. The outcome of SINA gave a multi-aligned *Streptomyces* sequences FASTA file which was processed with MEGA [38] to delete gap sites, and subsequently to run bootstrapped phylogenetic trees, using neighbor joining model.

### 2.4. Morphological Analysis

Three week-old plates of *Streptomyces* cultures on solid GYM medium were inspected and recorded under a stereo microscope (SZX16, Olympus, Japan), using a visual increase of 0.7-fold. Additionally, cells and spores of both *Streptomyces* strains were inspected under an Axiophot microscope using a 100x lens and recorded with Axio Cam MRm (Zeiss, Göttingen, Germany).

### 2.5. Chemical Analysis

After the growth period, 20 g/L of amberlite XAD-16 (Sigma-Aldrich, St. Louis, MO. USA) were added to each culture flask and mixed for one hour using orbital agitation in 120 rpm. Subsequently, the resin was separated through cheesecloth filtration [39], and the liquid was discarded. Amberlite was mounted on a glass funnel, and washed with 3 L of deionized water, and eluted with 1 L of acetone [39]. Acetone was then concentrated under reduced pressure until obtaining an aqueous residue. Subsequently, 1 L of deionized water was added to the acetone residue and brought to a separation funnel. To extract the organic molecules, 3 × 300 mL of ethyl acetate was used. The organic phase was concentrated under reduced pressure until dryness.

*Streptomyces* secondary metabolite profiles were acquired through high pressure liquid chromatography (HPLC, Merck-Hitachi, Darmstadt, Germany) coupled with evaporative light scattering detector (ELSD, Sedere, Olivet, France). The secondary metabolites profiling was developed primarily as screening strategy, using 30 min gradient. The gradient developed was as following: 0 min: 90% water, 10% acetonitrile, 20 min: 0% water, 100% acetonitrile, 23 min: 0% water, 100% acetonitrile, 28 min: 90% water, 10% acetonitrile, 30 min: 90% water, 10% acetonitrile. The used column was reverse phase C18 gravity SB™ (Macherey-Nagel, Düren, Germany). The wavelengths recorded were 210 and 254 nm. Dereplication process and 254 nm HPLC profiling were made with a High Resolution Liquid Chromatography coupled with Mass Spectroscopy (HRLCMS), Thermo Scientific™ UltiMate 3000 RS UHPLC coupled to a Thermo Scientific™ Q Exactive™ Hybrid-Quadrupol-Orbitrap MS (Thermo, Bremen, Germany), positive mode, and a 35 minutes gradient of H_2_O and acetonitrile supplemented with 0.1% of formic acid. The gradient developed was as following: 0 min: 90% water, 10% acetonitrile, 25 min: 0% water, 100% acetonitrile, 28 min: 0% water, 100% acetonitrile, 30 min: 90% water, 10% acetonitrile, 35 min: 90% water, 10% acetonitrile. The used column was reverse phase C18 gravity SB™ (Macherey-Nagel, Düren, Germany). The wavelength recorded was 254 nm. Mass and spectral data were evaluated with Xcalibur® (Thermo Scientific, San Jose, CA, USA), and compared with online databases (MarinLit, and Scifinder), and literature.

^1^H Nuclear Magnetic Resonance (NMR) experiments of the crude extracts were acquired to characterize the main components. The samples were dissolved in deuterated chloroform (Eurisotop™, Saint-Aubin, France), and transferred to NMR tubes (178 × 5.0 mm). Experiments were acquired on a Bruker Avance III spectrometer (Rheinstetten, Germany) operating at 600 MHz proton frequency equipped with a cryogenically cooled triple resonance z-gradient probe head using stand pulse sequences from the Bruker experiment library. Spectra were referenced against tetramethylsilane (TMS) as internal standard. NMR data was analyzed with TopSpin (version 3.5.b.91 pl 7, Bruker BioSpin Ltd., Karlsruhe, Germany).

### 2.6. Antibiotic Activity

The disc diffusion method was used to determine the antibiotic activity [40]. Thus, crude extracts obtained from the *Streptomyces* strains were tested to determine their activity on Gram-positive and Gram-negative bacteria. For this purpose, we chose *Staphylococcus lentus* DSM 20352, and *Escherichia coli* DSM 498. These bacteria were cultured in TSB medium (17 g of peptone from casein, 3 g peptone from soymeal, 2.5 g glucose × H_2_O, 5 g NaCl, 2.5 g K_2_HPO_4_, 1 L deionized water and pH to 7.3) at 37 °C for 24 h. Crude extracts were dissolved in MeOH to be subsequently transferred to a paper disc to reach a final concentration of 50 μg per disc. Additionally, we used an antibiotic susceptibility disc of streptomycin (Oxoid®, Columbia, MD, USA) as a positive indicator of antibiotic activity in a concentration of 25 μg per disc. All the used paper discs had a diameter of 6 mm. The plates were inoculated with fresh culture of *S. lentus* DSM 20352, and *E. coli* DSM 498, and incubated at 37 °C for 24 h. After the incubation period, the inhibition zone was measured and registered.

## 3. Results

### 3.1. Phylogenetic Analysis

Molecular characterization of both *Streptomyces* strains was performed with the sequence of the 16S rRNA gene as a genetic marker. The amplification and subsequent characterization resulted in nearly complete 16S rRNA sequences, whereby the Easter Island strain, *Streptomyces* sp. SN25_8.1, revealed a sequence with 1477 nucleotides (NCBI access# MK734066) compared to the sequence of the laboratory grown type strain of *Streptomyces griseus* subsp. griseus DSM 40236^T^, with a sequence of 1476 nucleotides (NCBI access# MK734067). We used the new sequence of the 16S rRNA gene of the type strain, *Streptomyces griseus* subsp. griseus DSM 40236^T^, for detailed sequence comparison, since the publicly available sequence dates to 2003 (NCBI access# AY207604). Both the new and old sequences are identical.

**Figure 1:**
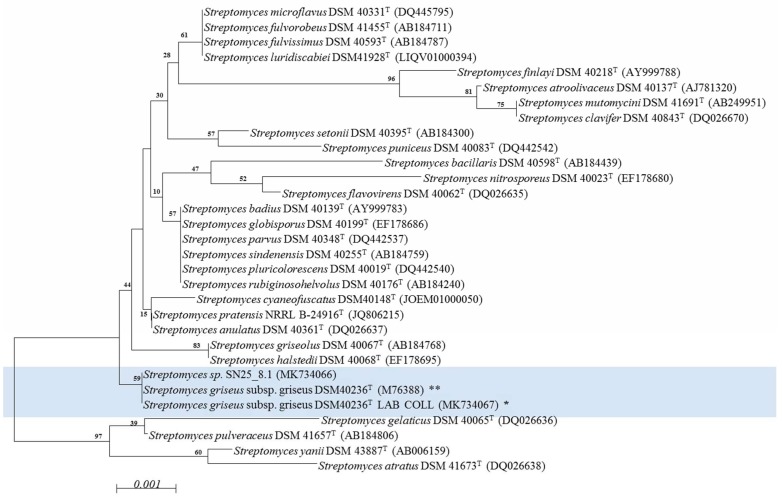
Phylogenetic characterization of the *Streptomyces* strains using a neighbor joining model. Light blue: experimentally compared *Streptomyces* strains. Bootstrap = 1000; Bootstrap values are shown on the branch, where 100 is maximum; ^T^: type strain; NCBI access number is within parenthesis. * *Streptomyces griseus* subsp. griseus DSM 40236^T^ LAB COLL: Sequence experimentally obtained from fresh cultures and deposited in NCBI (MK734067). ** *Streptomyces griseus* subsp. griseus DSM 40236^T^: Sequence retrieved from NCBI (M76388). The evolutionary distances were computed using the Jukes-Cantor method and are in the units of the number of base substitutions per site (scale). The analysis involved 31 nucleotide sequences. All positions containing gaps and missing data were eliminated. There were a total of 1338 positions in the final dataset.

The alignment of the 16S rRNA gene sequences of strains DSM 40236^T^ (from a Russian garden soil sample) and SN25_8.1 (from Easter Island) showed that the strains shared identical sequences of the 16S rRNA gene (Supplementary Figure S1). The phylogenetic tree (Figure 1) revealed, that both strains affiliate to one cluster, which is separated from the other *Streptomyces* spp. strains.

### 3.2. Morphological Comparison

**Figure 2:**
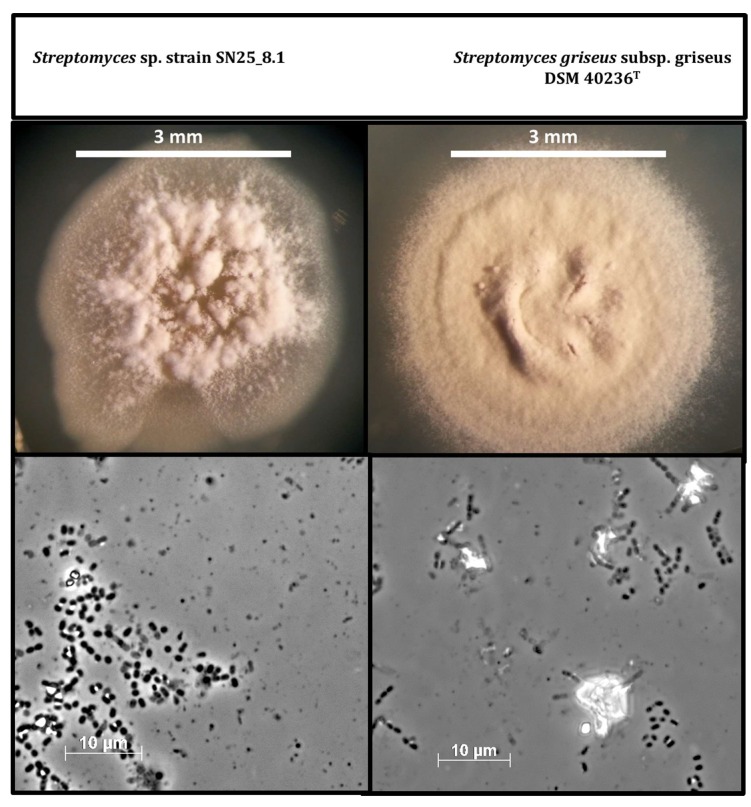
Comparison of cell and colony morphology of the two *Streptomyces* strains after growth on GYM medium for three weeks. Photos were recorded using a stereo microscope (upper figures) and microscope with a 100x lens (bottom figures).

After cultivation of the strains in solid GYM medium for three weeks, we continued with a morphological comparison of the Easter Island *Streptomyces* sp. SN25_8.1 and the type strain, *Streptomyces griseus* subsp. *griseus* DSM 40236^T^ (Figure 2). The visual comparison showed evident macroscopic differences between the strains, such as; spore pigmentation, amount of spore formation, and aerial hyphae distribution on the colony. Light microscopic examination of the cells and spores of both strains showed no differences. Nevertheless, a detailed characterization of the spores and spore-bearing hyphae would require scanning electron microscopy.

### 3.3. Secondary Metabolites Production

**Figure 3:**
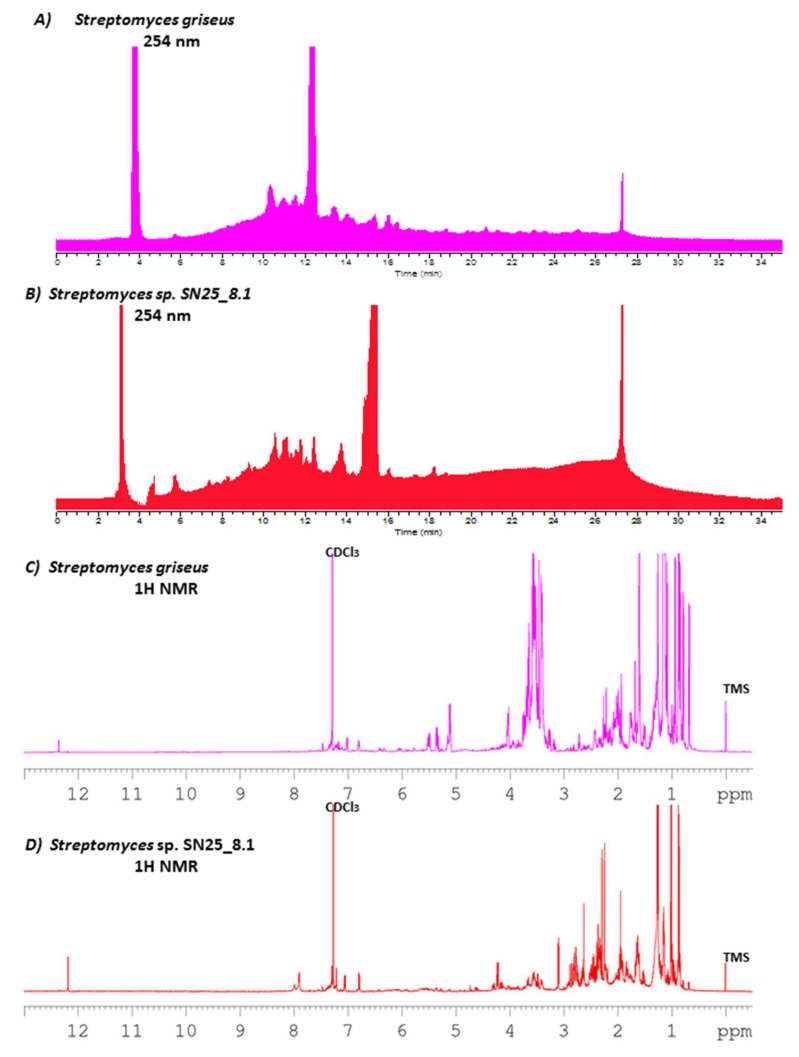
HPLC metabolite profile comparison of *Streptomyces* sp. SN25_8.1 from Easter Island, and *Streptomyces griseus* subsp. *griseus* DSM 40236^T^ from Russia, measured at 254 nm (**A**,**B**), and ^1^H NMR comparison of the crude extract of both strains (**C**,**D**). CDCl_3_: deuterated chloroform; TMS: tetramethylsilane.

Both *Streptomyces* strains produced different metabolites patterns and quantities after growth in a Starch-Glucose-Glycerol medium (slightly modified SGG). *Streptomyces* sp. SN25_8.1 produced a total of 60.9 mg of crude extract, while the type strain *Streptomyces griseus* subsp. *griseus* DSM 40236^T^ produced almost the double amount, 108.1 mg. The HPLC chromatograms of the crude extracts showed differences and similarities between the two *Streptomyces* strains (Figure 3A,B). The similarities found in the chromatograms (taking the Easter Island representative as a point of comparison, Figure 3B) were observed in the following retention time (RT): 5.8, 8.5, 9.33, 9.54, 11.0, 11.5, 12.0, 12.4, 13.1, 15.3, 16.0 and 18.8 min. The observed peak at RT 27.3 min is a methodic artifact and should not be considered in the comparison of the chromatograms. Differences were found between the chromatograms at RT 3.1, 4.5, 5.3, 7.2, 11.3, 11.6, 13.7, and 18.46. Considering this information, it is fair to state that these *Streptomyces* strains differ significantly, because they share some metabolites but differ in the production of others.

In order to extend the previous HPLC data, ^1^H NMR experiments were performed to again display similarities as well as differences among the most abundant chemical groups present in both samples (Figure 3C,D). For example, remarkable similarities were found in the aromatic proton zone (7–8 ppm), in the olefinic proton zone (of 4–6, ppm), and in the low frequency region (1.0–3.5 ppm), where aliphatic methyl, methylenes and methines are commonly found. However, abundant differences between the two spectra are apparent for example in the high frequency protons around 12.2–12.4 ppm, the broad peaks close to 8 ppm, olefinic signals around 5.5 ppm, and the methyl protons around 0.65 ppm. Further, in particular the strongly differing intensities in the mentioned regions suggest also the presence of large differences in the relative metabolite contributions in these mixtures.

**Figure 4:**
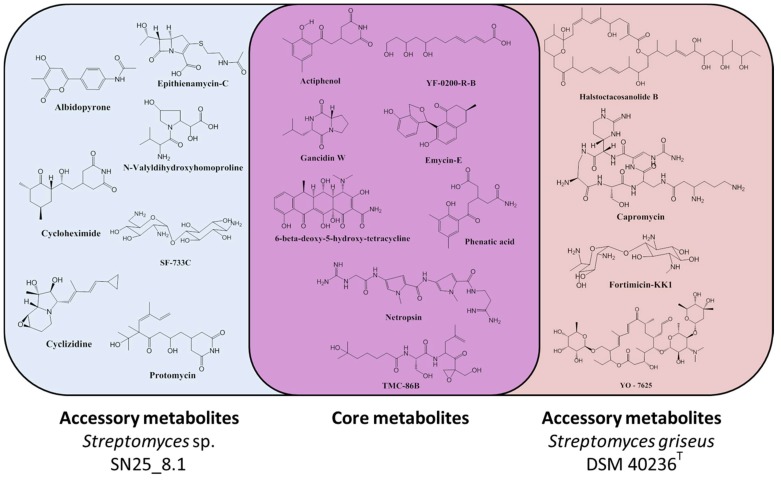
Chemical diversity of the studied *Streptomyces* representatives. Core metabolites: Metabolites shared by both strains. Accessory metabolites: Unique metabolites for each of the studied strains.

As simple one-dimensional ^1^H NMR profiles do not allow for a compound identification, attempts to identify some of the mixture components by high resolution LC-MS techniques (HRLCMS) were undertaken. Several molecules could be identified in both *Streptomyces* representatives based on their exact masses and a chemical database search (Supplementary Materials). We observed that the two *Streptomyces* strains had a core chemical arsenal that is shared by both strains and an accessory chemical arsenal which seems to be strain specific (Figure 4). The identified core chemicals comprised gancidin W, YF-0200-R-B, emycin E, phenatic acid, netropsin, actiphenol, 6-beta-deoxy-5-hydroxy-tetracycline and TMC-86B. From the analysis of the accessory metabolites, we observed that the Easter Island representative, *Streptomyces* sp. SN25_8.1, produced seven detectable chemicals, which were: albidopyrone, cyclizidine, epithienamycin C, cycloheximide, SF-733C, protomycin and N-Valyldihidroxyhomoproline, as accessory metabolites. In contrast, we could detect only four chemicals in the reference strain *Streptomyces griseus* subsp. griseus DSM 40236^T^ as accessory metabolites, which were: fortimicin KK1, capromycin, YO-7625 and halstoctacosanolide B.

It is evident that the identified metabolites may be only a fraction of the total chemical components in the crude extracts. However, HRLCMS allowed us to have a depiction of the chemical diversity of both *Streptomyces* strains under the same analytical conditions.

The major components were determined through the use of HPLC-ELSD. The reference strain, *Streptomyces griseus* subsp. griseus DSM 40236^T^, showed two main components: phenatic acid (RT 12.3 min), and a second one with RT 3.8 min, which could not be identified due to the lack of ionization in HRLCMS measurement. The major components in the Easter Island strain, *Streptomyces* sp. SN25_8.1, were determined as cycloheximide (RT 11.33 min) and actiphenol (RT 15.3 min).

Interestingly, *Streptomyces griseus* strains are well known for producing the antibiotic streptomycin, but this molecule was not produced by the strains under our experimental condition. In both *Streptomyces* strains, we found chemicals which did not show any match to known *Streptomyces* metabolites, suggesting potentially novel molecules. In the case of the Easter Island *Streptomyces* strain, we observed that the unknown chemical had a molecular weight of [M+H]^+^ m/z 579.53381. This chemical showed a polyprotonation pattern, which points towards a peptide structure. The reference strain, *Streptomyces griseus* subsp. griseus DSM 40236^T^ showed an unknown metabolite with a molecular weight of [M+H]^+^ m/z 813.59229. The isolation of these compounds from the crude extracts and chemical analyses would be necessary for structure elucidation.

### 3.4. Antibiotic Activity

Antibiotic activity of the two *Streptomyces* crude extracts (Table 1) were evaluated through disc diffusion assay, using Gram-positive and Gram-negative bacteria. For the assay, we selected *Staphylococcus lentus* DSM 20352 and *Escherichia coli* DSM 498.

As shown in Table 1, the antibiotic activities of the crude extracts of both *Streptomyces* strains were quite similar as both produced an inhibition zone with the Gram-positive and the Gram-negative bacterium. Importantly, the crude extract of the reference strain, *Streptomyces griseus* subsp. griseus DSM 40236^T^, had a stronger inhibitory effect against the Gram-negative bacterium, *E. coli*, which may be related to the higher concentrations of its components. The dereplication experiment suggested the presence of several metabolites with antibiotic activity like 6-beta-deoxy-5-hydroxy-tetracycline, gancidin W, phenatic acid and netropsin, which may be responsible for the observed antibiotic activity to both Gram-positive and Gram-negative bacteria. Streptomycin was used as a positive control.

## 4. Discussion

Much effort is being made for the isolation of new biologically active *Streptomyces* strains [41], since this genus has been found to be a reliable source of chemicals with human health application [42]. However, a significant number of new *Streptomyces* isolates are affiliated through the 16S rRNA molecular marker to already described species [43], a reason why they are also considered to be identical in secondary metabolite production.

Recent studies have proposed alternative analyses to determine the dissimilarity of closely related *Streptomyces* strains, such as: polyphasic characterization, multilocus sequence typing and full genome sequencing [19,44], which have shown to be a more precise tool for phylogenic clarification and secondary metabolite dereplication [45,46,47,48]. None of these studies have dealt with the aspect of the large-scale geographic separation of the *Streptomyces* strains, which was investigated.

Alternatively, a direct comparison through the laboratory growth of *Streptomyces* still remains a more affordable and rapid way of determining how similar the metabolite profiles of the two strains are. Once grown, the studied *Streptomyces* can be compared using HRLCMS and ^1^H NMR techniques, which vastly help in the dereplication process, since they are quite informative about the chemical identities and functionalities of the crude extracts.

Our results showed that *Streptomyces* closely related through the 16S rRNA gene marker presented differences in macroscopic features (pigmentation, aerial hyphae distribution, colony morphology), but kept similarities in cell morphology. In terms of secondary metabolite production, we found that closely related *Streptomyces* species kept a common set of chemicals, which has been addressed as core secondary metabolites [49]. This fact is in agreement with the common knowledge that *Streptomyces* strains with similar 16S rRNA gene sequences produce identical chemicals [44]. However, our finding also indicated that *Streptomyces* strains have a set of accessory secondary metabolites that are unique for each isolate, despite of the identical 16S rRNA gene sequences. These findings have also been observed in other *Streptomyces* species. For example, Antony-Babu et al. [19], developed a polyphasic analysis of 10 different *Streptomyces* strains with identical 16S rRNA gene sequences and they found, that by evaluating characteristic features like halotolerance, optimal pH growth, coloration and GC content at least 5 out 10 clearly diverged as new species. These results were further supported with a multilocus sequence analysis and phylogenetic tree. This study also showed the production of a core set of secondary metabolites and an accessory chemical diversity.

Another example was provided by Vicente et al. [44], who performed a similar study, but evaluated the genomes of six closely related *Streptomyces* strains. The evaluation of the biosynthetic gene clusters showed that the analyzed *Streptomyces* strains kept a core set of secondary metabolites and in addition, a set of strain-specific metabolites. Interestingly, Vicente et al. [44] also reported a set of chemicals with wide presence in *Streptomyces*, such as melanin, desferrioxamine B, hopene, isorenieratene and geosmin. However, none of them could be detected in our experimental work. This observation might be related with the evolutionary distance between *S. griseus* strains and the *Streptomyces* strains used by Vincente et al. [44]. This last finding may also be an indication of species-specific metabolites. Interestingly, Vicente et al. [44] also reported chromosome reorganization events (pericentral inversion) and suggested horizontal gene transfer for the acquisition of biosynthetic gene clusters for strain-specific secondary metabolites. Finally, this study also conveyed the finding of unknown biosynthetic gene clusters, suggesting potential for novel chemistry production.

Our data indicated that *Streptomyces* strains of the same species, isolated from geographical distant locations, can show important differences in the metabolite profiles. These may be overlooked if solely a genetic marker such 16S rRNA gene sequence is used as an indicator of secondary metabolite diversity. As described previously, genome sequencing and comparison, multilocus gene analysis or growth experiments associated with chemical analysis are three suitable alternatives to dereplicate closely related *Streptomyces* strains. This strategy can represent a valuable source of metabolites with biomedical and industrial application, by preventing the discard of unstudied *Streptomyces*.

Secondary metabolite production in *Streptomyces* is not essential for their life cycle, however these metabolites confer an evolutionary advantage over competitors, since these molecules can be used as a chemical weapon to control other bacterial and fungal competitors (deterrence, inhibition, decease). Since *Streptomyces* can profit from these molecules, *Streptomyces* might adapt their chemical arsenal in function of their habitat and their competitors to succeed in new environments.

It remains an open but interesting question, how genes necessary for the production of a particular secondary metabolite have been gained by individual *Streptomyces* strains. Different researchers have discussed this process in other actinobacterial genera, and the most widely accepted hypothesis is the horizontal transfer of genes (HGT) of entire biosynthetic pathways [50], which may have direct relation to the environment in which the *Streptomyces* strains are dwelling. However, gene duplication, mutation and genetic rearrangement should also not be discarded in the process of modifying secondary metabolite biosynthetic pathways. The gain and loss of secondary metabolites may be considered as a biochemical evolution of *Streptomyces* strains, since the process of selecting and discarding genes for the production of secondary metabolites may have direct relation to the environmental competition and survival needs of the strain. It seems reasonable to assume that phylogenetic almost identical *Streptomyces* strains may have diverged from a common ancestor because of their genetic and chemical similarities. Within a sufficient timeframe, *Streptomyces* strains may have acquired new biosynthetic abilities to produce different secondary metabolites, because of the need to adapt and specialize in their particular ecological niche. Environmental pressure may be a driving force for the retention/discarding/acquisition of the secondary metabolite genes. It has been shown that *Streptomyces* strains are capable of spontaneous combination of genetic information [21,51], generating previously unknown chemical hybrids [52]. However, it is not known for how long these naturally occurring *Streptomyces* hybrids have existed in the environment since many factors may influence the success of the new ecotypes.

## 5. Conclusions

We established that 16S rRNA gene sequences do not provide information reliable enough to evaluate the chemodiversity of *Streptomyces* strains since our analyses of the metabolite profiles showed differences in the production of secondary metabolites of strains identical on the basis of this genetic marker. While we found a core set of secondary metabolites that is identical in both strains, a set of even more diverse accessory metabolites appear to be strain specific. Based on the phylogenetic closeness and the similarity of the metabolites, it is suggested that both *Streptomyces* had a common origin which went through a subsequent specialization in function of their habitat. In conclusion, this study has demonstrated that *Streptomyces* strains, with an identical phylogenetic classification to already known strains, still represent a diverse and putative source of novel secondary metabolites with potential for drug discovery; therefore, they should not be discarded in screening processes for bioprospection.

## Figures and Tables

**Table 1:** Antibiotic activity of crude extracts.

Sample Tested	Inhibition Zone (mm)	
	*S. lentus* DSM 20352	*E. coli* DSM 498
*Streptomyces* sp. SN25_8.1	8	15
*Streptomyces griseus* subsp. *griseus* DSM 40236^T^	8	20
Streptomycin	18	20

## Supplementary Materials

The following are available online at https://www.mdpi.com/2076-2607/7/6/166/s1, Figure S1: 16S rRNA gene sequence alignment of the *Streptomyces* strains. Supplementary materials also contains dereplication overview, the individual identification of the molecules, their structures and chemical data, as well as antibiotic activity of the crude extracts.
