# Supplementary material for: Different Secondary Metabolite Profiles of Phylogenetically almost Identical Streptomyces griseus Strains Originating from Geographically Remote Locations
Source: Microorganisms. 2019 Jun 6;7(6):166. doi: 10.3390/microorganisms7060166 (PMC6616549; doi:10.3390/microorganisms7060166)
Supplement: Supplementary file 1 [file microorganisms-07-00166-s001.pdf]

microorganisms

MDPI

Article

Different secondary metabolite profiles of phylogenetically almost identical *Streptomyces griseus* strains originating from geographically remote locations

Ignacio Sottorff[1, 2], Jutta Wiese[1], Matthias Lipfert[3], Nils Preußke[3], Frank D. Sönnichsen[3], and Johannes F. Imhoff[1,*]

[1] GEOMAR Helmholtz Centre for Ocean Research Kiel, Marine Microbiology, 24105 Kiel, Germany; isottorff@geomar.de, jwiese@geomar.de, jimhoff@geomar.de

[2] Facultad de Ciencias Naturales y Oceanográficas, Universidad de Concepción, 4070386 Concepción, Chile.

[3] Otto Diels Institute for Organic Chemistry, University of Kiel, 24118 Kiel, Germany; fsoennichsen@oc.uni-kiel.de, mlipfert@oc.uni-kiel.de, npreusske@oc.uni-kiel.de * Correspondence: jimhoff@geomar.de; Tel.: +49 431 600-4450

Table of contents

Alignment of 16S rRNA 1
Summary table of *Streptomyces* dereplication-HRLCMS 2
Supplementary material: dereplication-HRLCMS 3
Dereplication-HRLCMS of *Streptomyces* SN25_8.1 4
- Albidopyrone 5
- Cyclizidine 6
- Gancidin W 7
- YF-0200-R-B 8
- Emycin E 9
- 6-beta-deoxy-5-hydroxy-tetracycline 10
- Epithienamycin C 11
- SF-733 C 12
- Cycloheximide 13
- Phenatic acid 14
- Netropsin 15
- N-Valyldihidroxyhomoproline 16
- Actiphenol 17
- TMC-86B 18
- Protomycin 19
- Unknown 20

Dereplication-HRLCMS of *Streptomyces griseus* subsp. griseus DSM 40236T 21
- Gancidin W 22
- YF-0200-R-B 23
- Emycin E 24
- 6-beta-deoxy-5-hydroxy-tetracycline 25
- Fortimicin KK1 26
- Phenatic acid 27
- Netropsin 28
- Actiphenol 29
- TMC-86B 30
- Capromycin 31
- Halstoctacosanolide B 32
- YO-7625 33
- Unknown 34

Streptomyces griseus 16S rRNA comparison 35
Phylogenetic results: *Streptomyces* sp. SN25_8.1 36
Phylogenetic results: *Streptomyces* griseus subsp. griseus DSM 40236T 42
Antibiotic test 48
HPLC-ELSD Abundance profile for *Streptomyces* sp. SN25_8.1 49
HPLC-ELSD Abundance profile for *Streptomyces griseus* subsp. griseus DSM 40236T 50

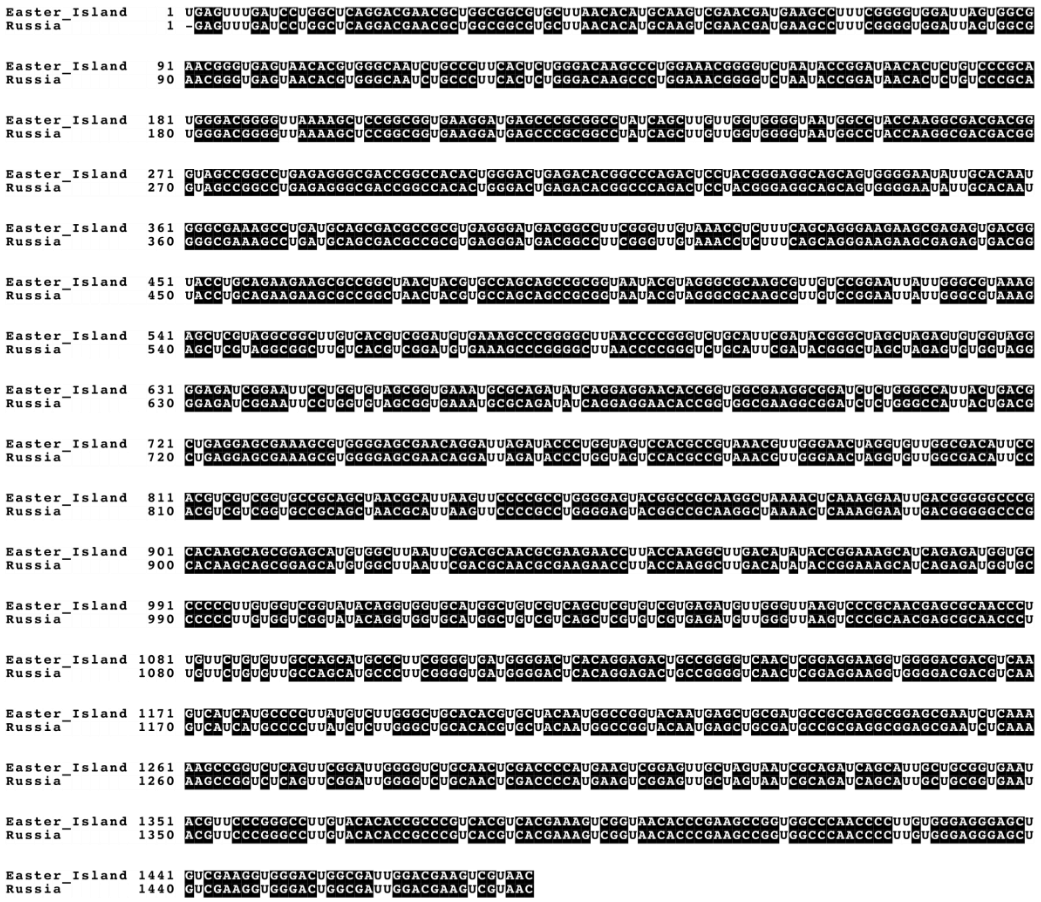

Supplementary Figure 1 Alignment of the 16S rRNA gene sequences of two *Streptomyces* strains. Easter Island: *Streptomyces* sp. SN25_8.1 (NCBI access# MK734066), Russia: *Streptomyces griseus* subsp. griseus DSM 40236^{T} (NCBI access# MK734067)

Dereplication - HRLCMS

Table 1 Dereplication overview

Streptomyces sp. SN25_8.1			*Streptomyces griseus* subsp. griseus DSM 40236 T		
rt	mass	compounds	rt	mass	compounds
3.1	259.0844	Albidopyrone			
5.38	292.19019	Cyclizidine			
8.58	211.14388	Gancidin W	8.58	211.14388	Gancidin W
9.33	245.12811	YF-0200-R-B	9.33	245.12811	YF-0200-R-B
9.54	311.13843	Emycin E	9.54	311.13843	Emycin E
10	445.23267	Tetracycline	10	445.23267	Tetracycline
10.56	315.13336	Epithienamycin C			
			10.65	367.26843	Fortimicin KK1
11.33	282.16956	Cycloheximide			
11.6	340.1748	SF-733 C			
12.39	294.13306	Phenatic acid	12.39	294.13306	Phenatic acid
13.12	431.20593	Netropsin	13.12	431.20593	Netropsin
13.73	261.18454	N-Valyldihidroxyhomoproline			
15.3	276.12268	Actiphenol	15.3	276.12268	Actiphenol
17.26	415.21118	TMC-86B	17.26	415.21118	TMC-86B
18.46	352.2164	Protomycin			
			18.97	669.47778	Capromycin
			19.18	831.60242	Halstoctacosanolide B
			20.42	889.64447	YO-7625
			24.98	813.59229	Unknown
24.8	579.53381	Unknown			

Supplementary material

Dereplication - HRLCMS

Streptomyces sp. SN25_8.1

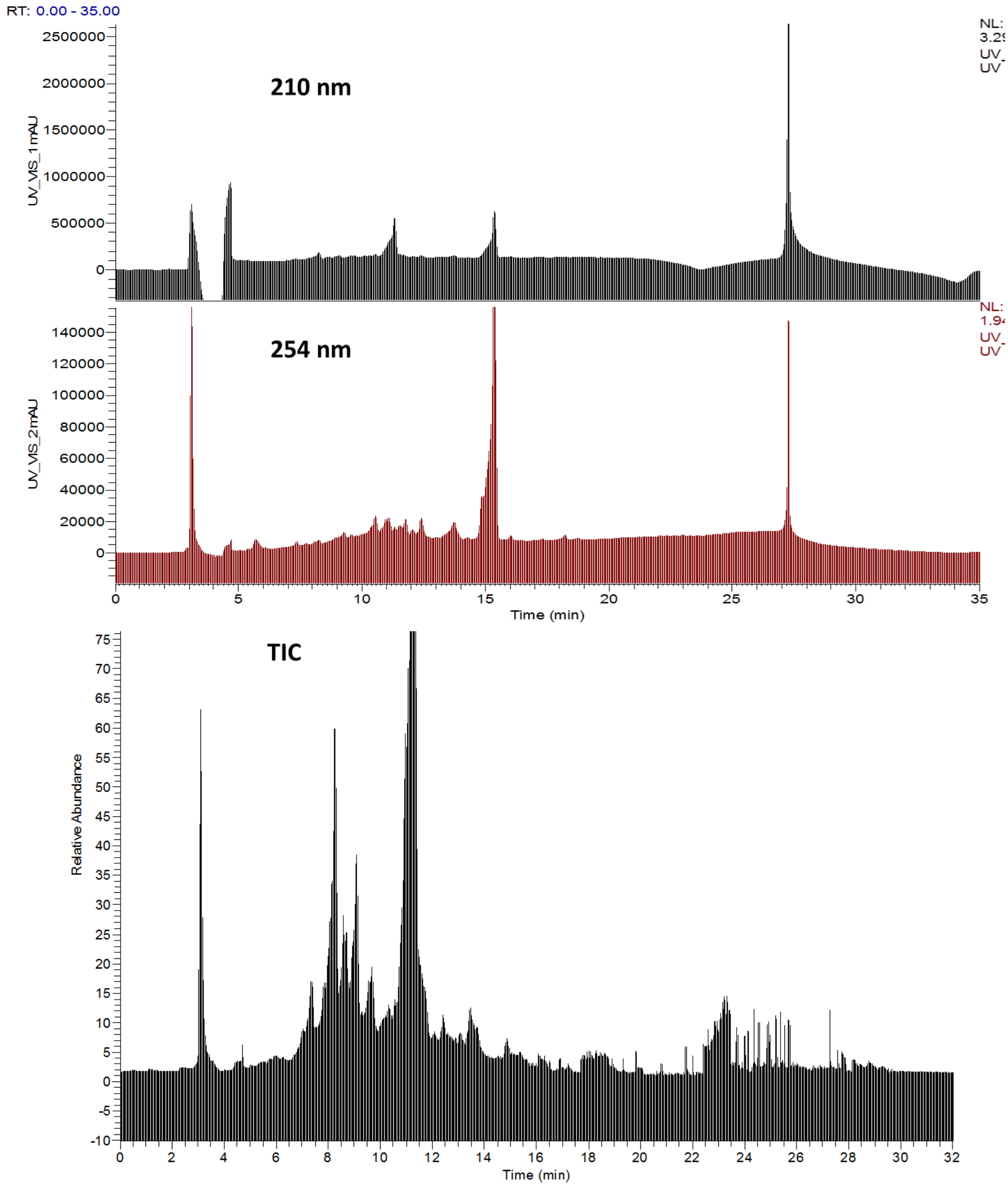

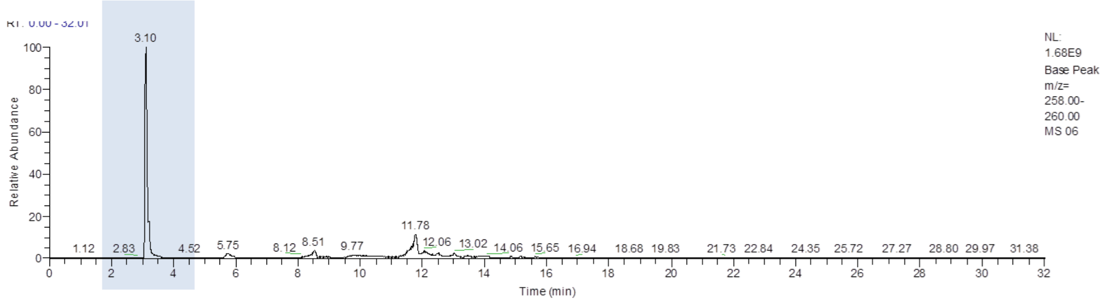

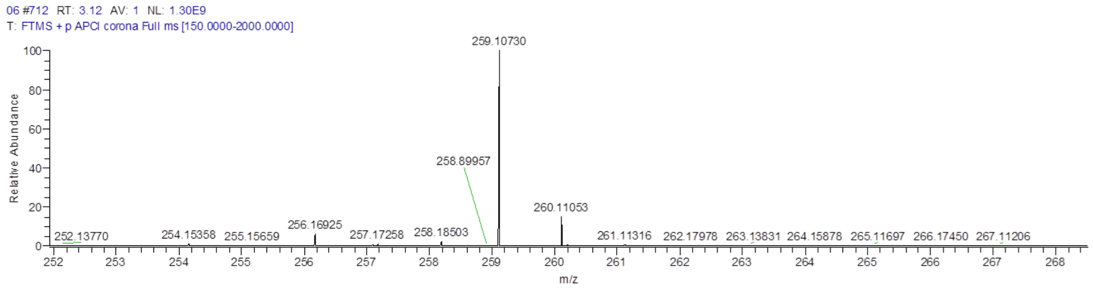

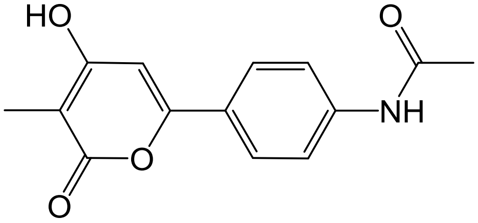

Albidopyrone

Chemical Formula: $C_{14}H_{13}NO_4$

Exact Mass: 259.08

Albidopyrone

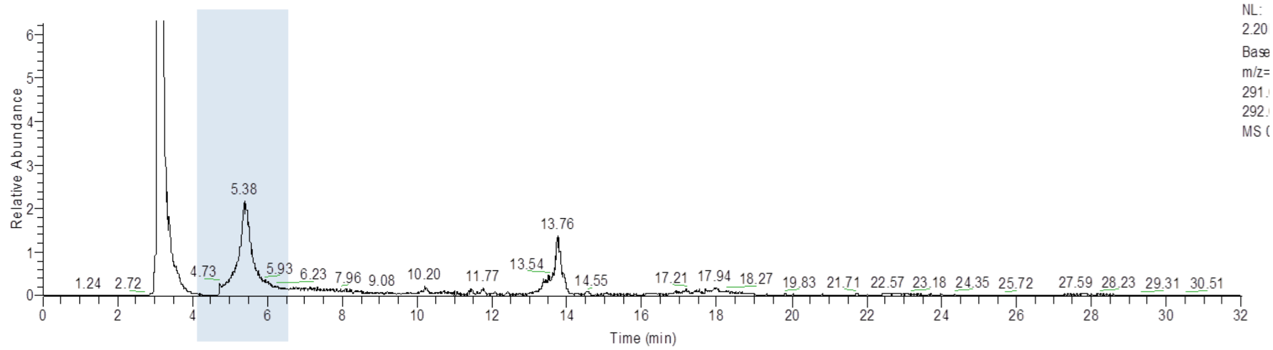

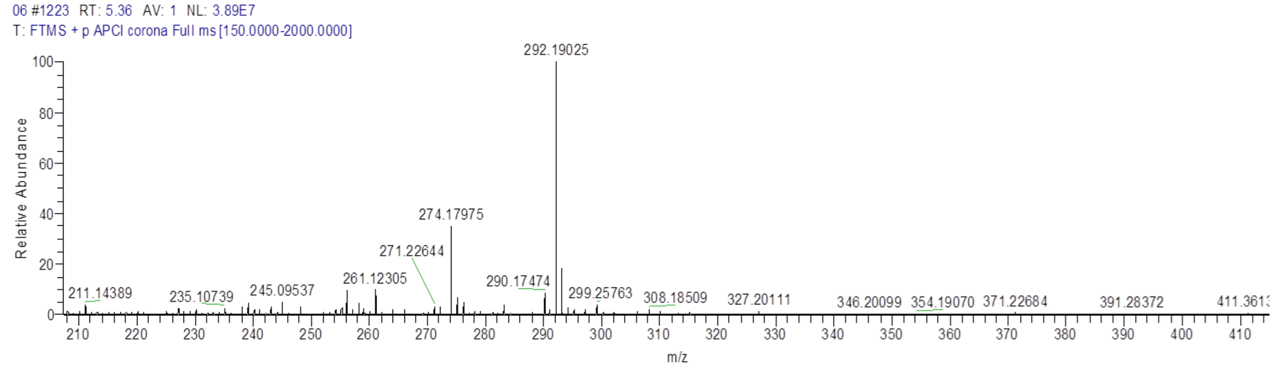

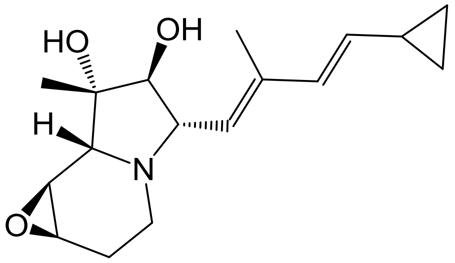

Cyclizidine

Chemical Formula: $C_{17}H_{25}NO_3$

Exact Mass: 291.18

Cyclizidine

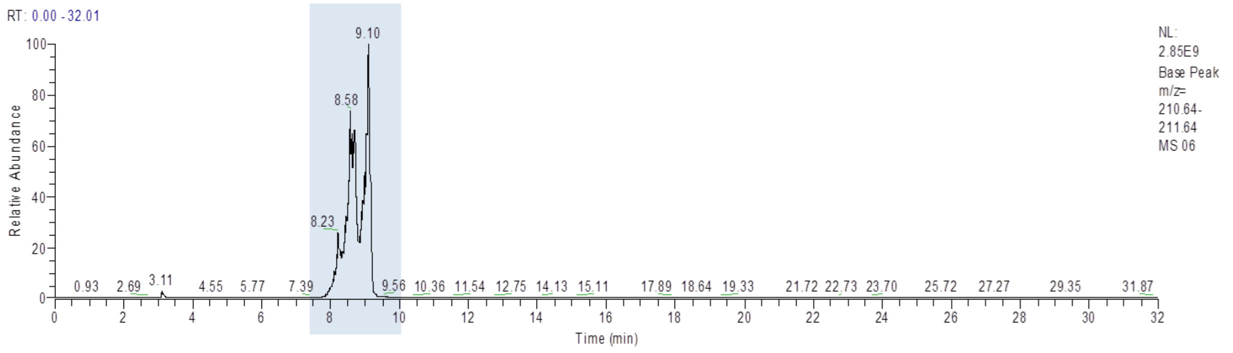

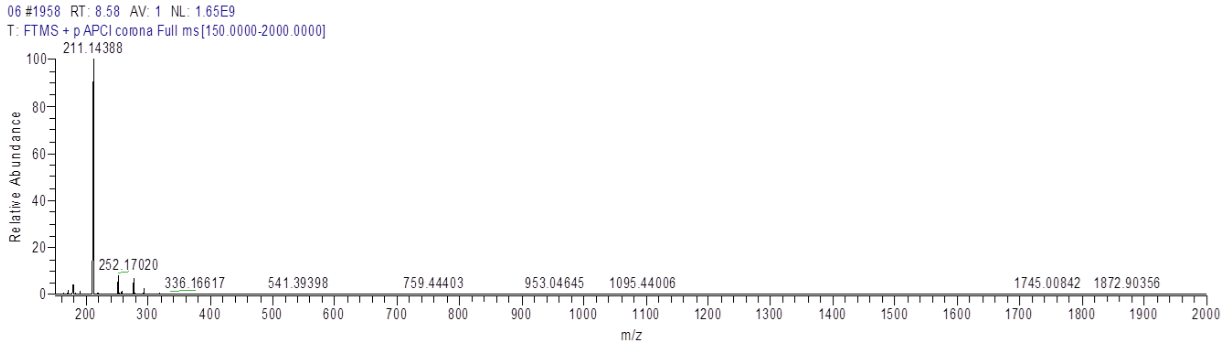

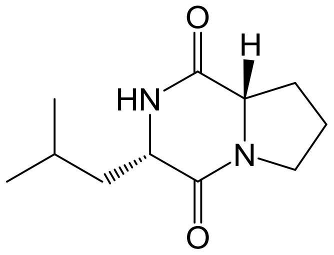

Gancidin W

Chemical Formula: $C_{11}H_{18}N_2O_2$
Exact Mass: 210.14

Gancidin W

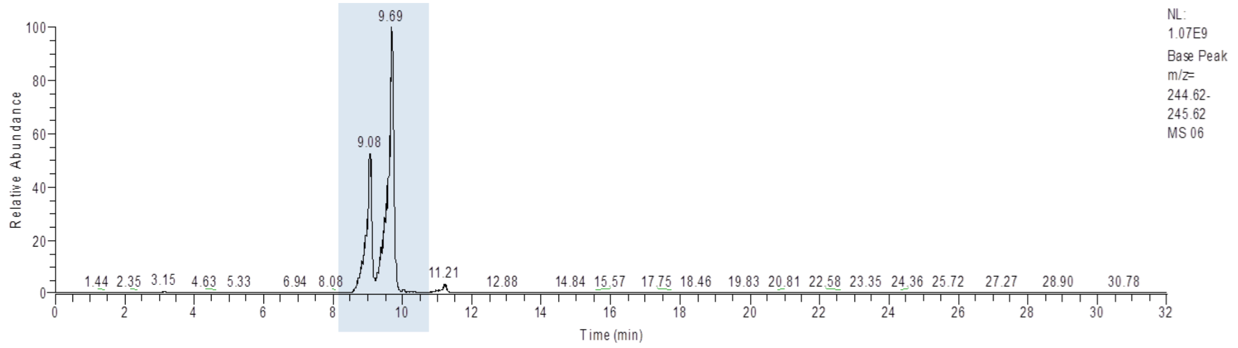

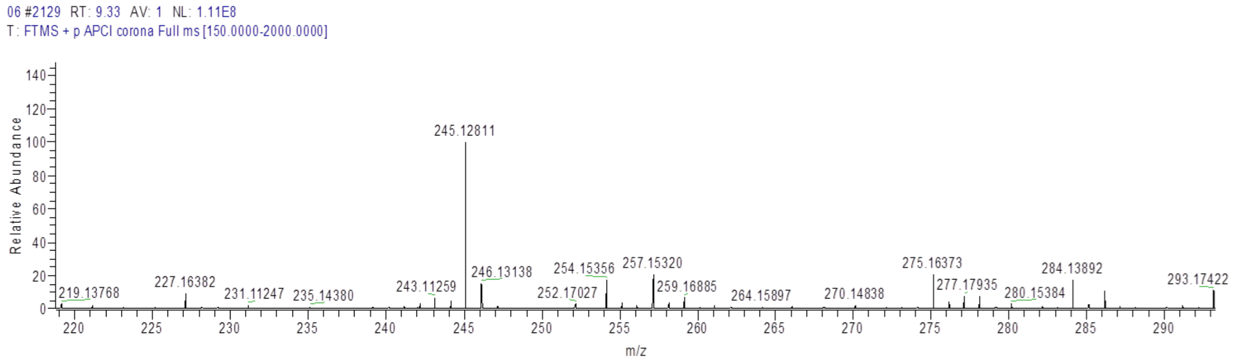

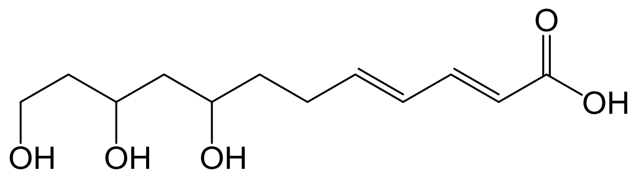

YF-0200-R-B

Exact Mass: 244.13
Molecular Weight: 244.29

YF-0200-R-B

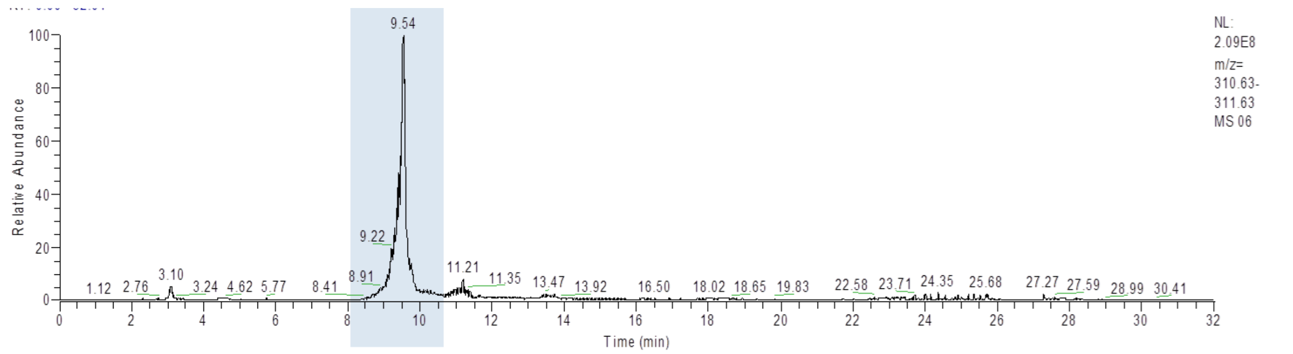

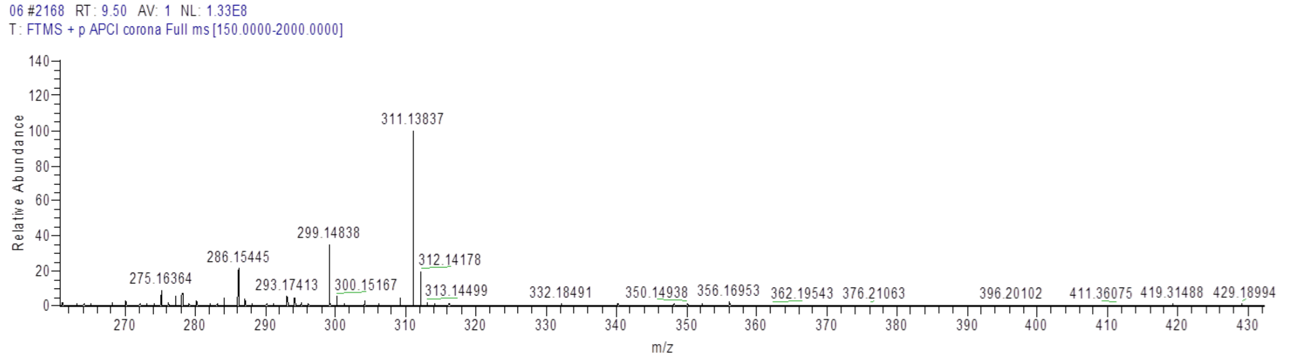

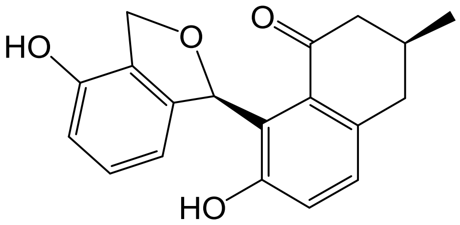

Emycin-E

Chemical Formula: $C_{19}H_{18}O_4$
Exact Mass: 310.12

Emycin E

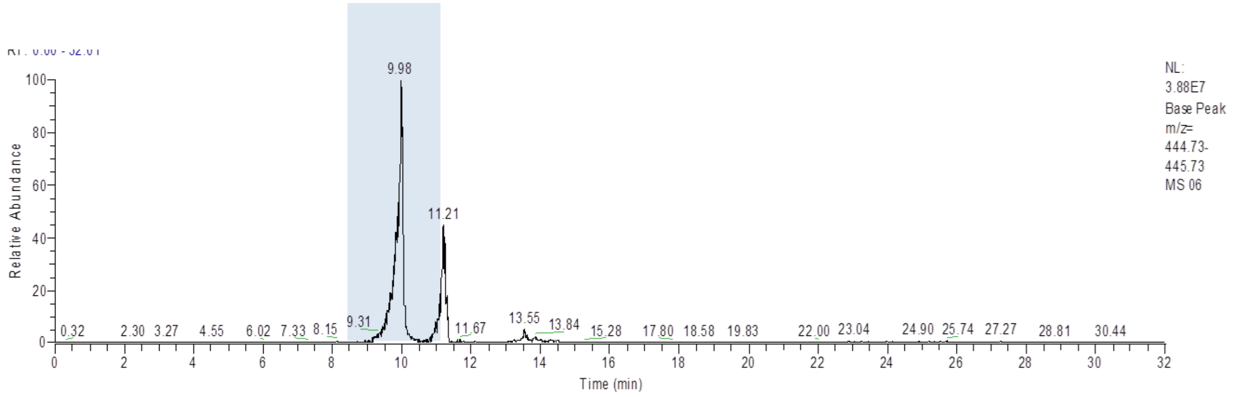

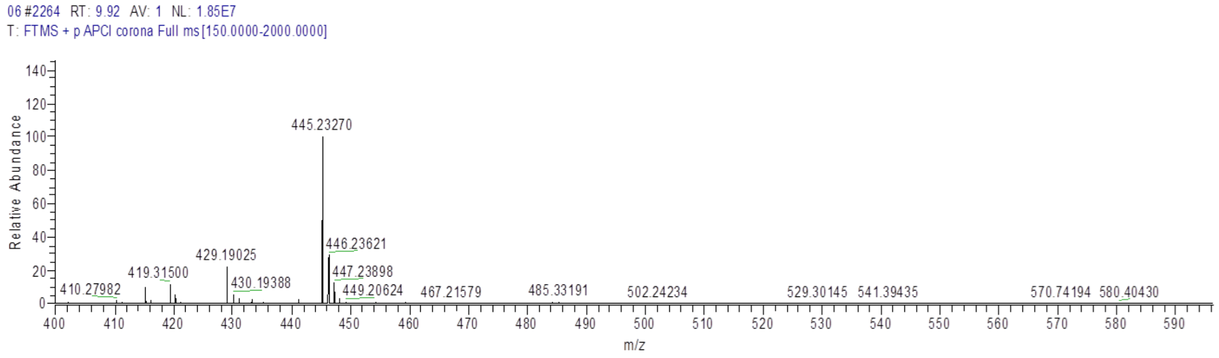

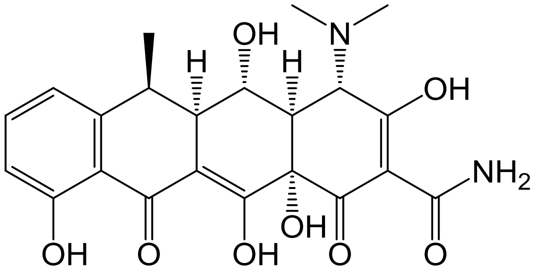

6-beta-deoxy-5-hydroxy-tetracycline

Chemical Formula: $C_{22}H_{24}N_2O_8$
Exact Mass: 444.15

6-beta-deoxy-5-hydroxy-tetracycline

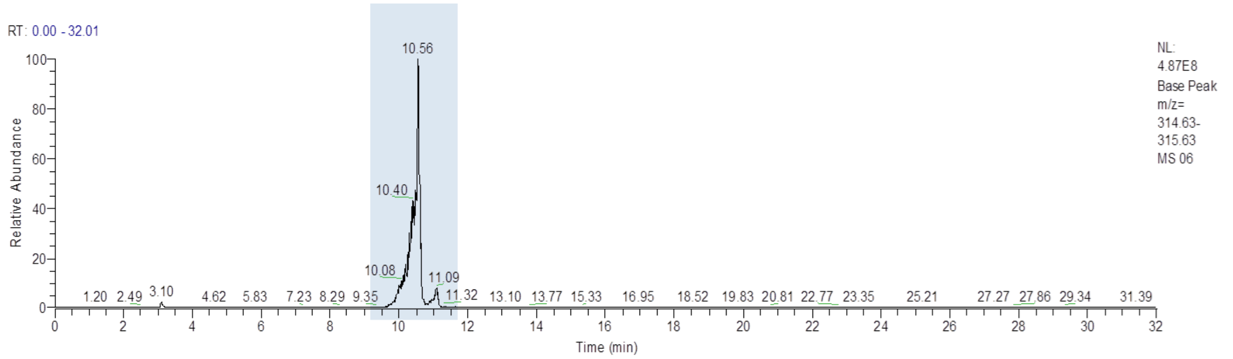

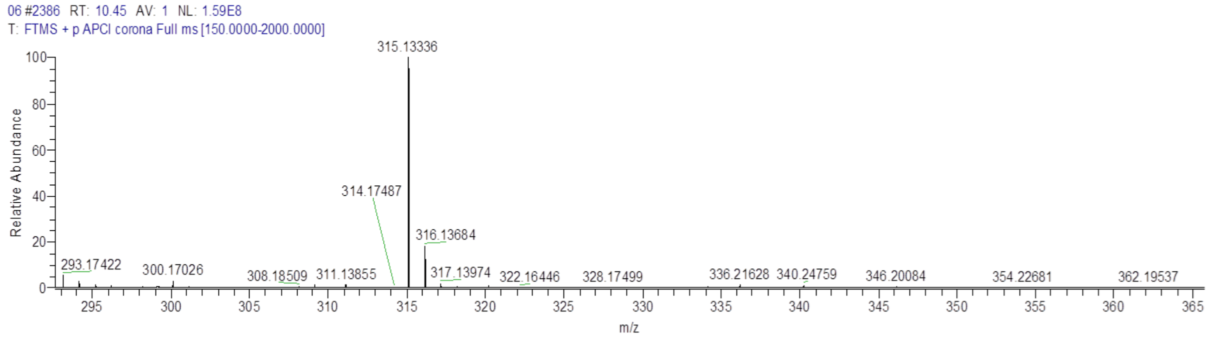

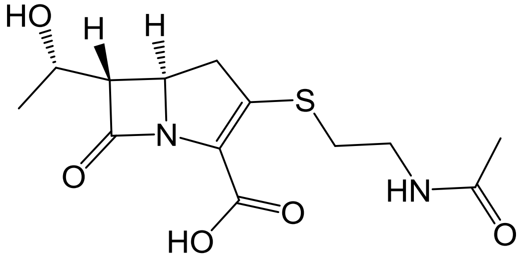

Epithienamycin-C

Chemical Formula: $C_{13}H_{18}N_2O_5S$
Exact Mass: 314.09

Epithienamycin C

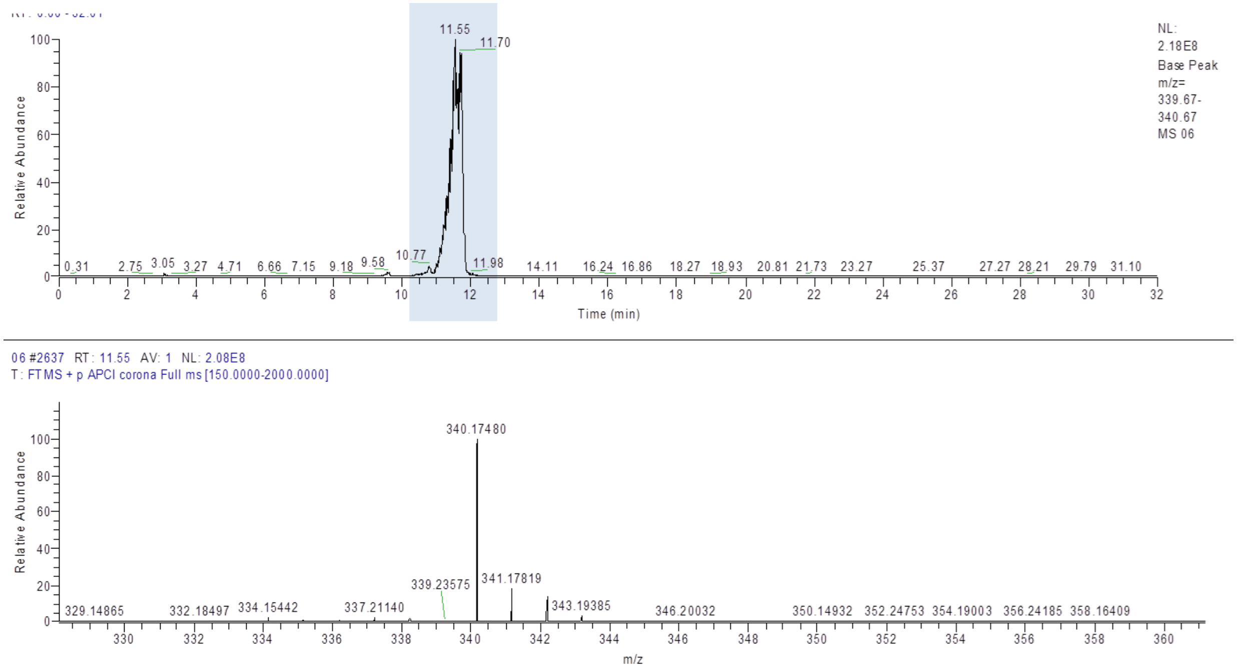

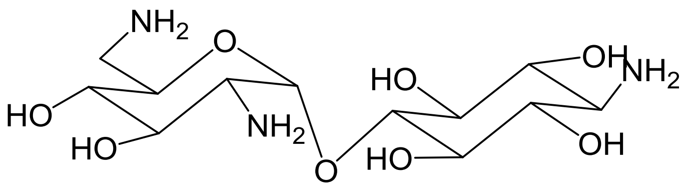

SF-733C

Chemical Formula: $C_{12}H_{25}N_3O_8$

Exact Mass: 339.16

SF-733 C

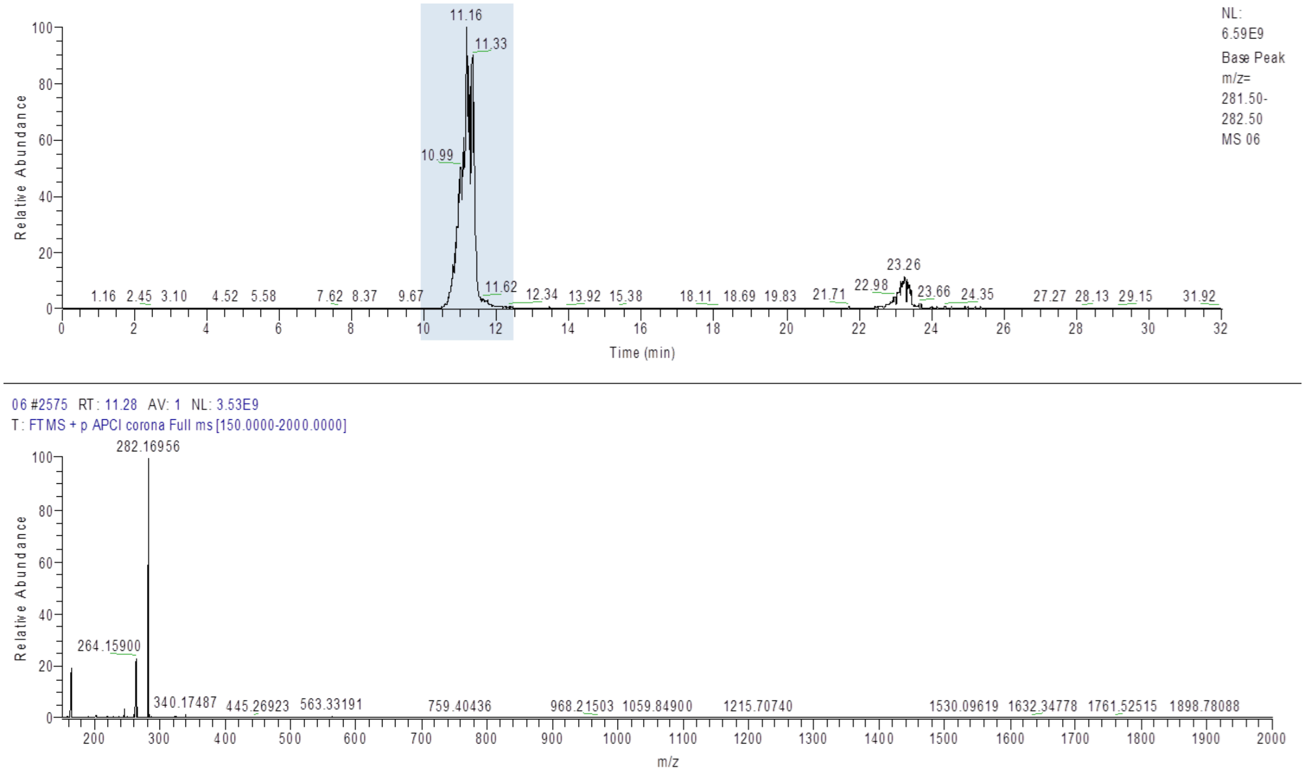

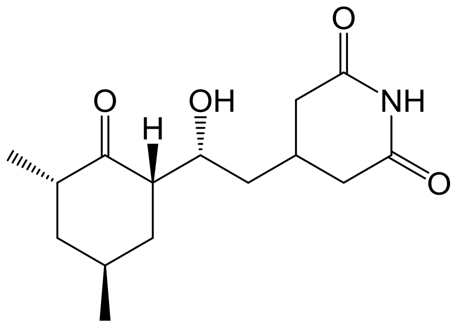

Cycloheximide

Chemical Formula: $C_{15}H_{23}NO_4$

Exact Mass: 281.16

Cycloheximide

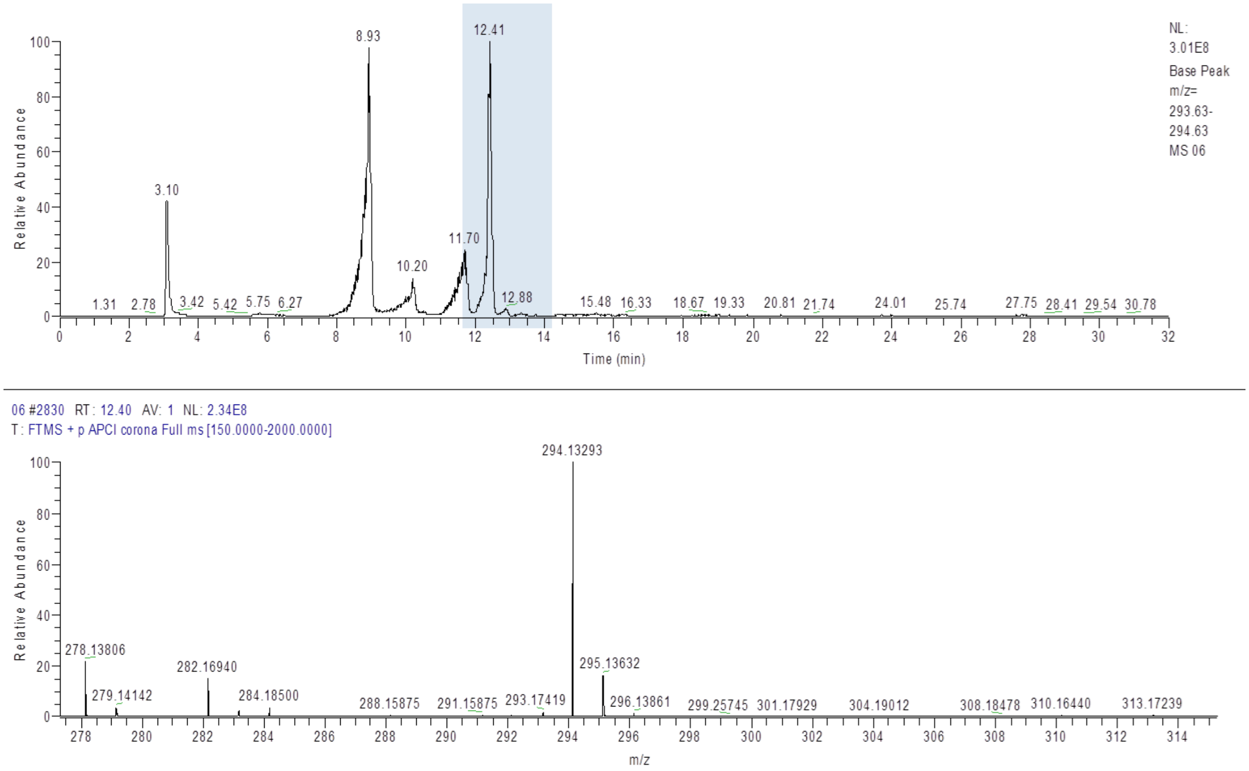

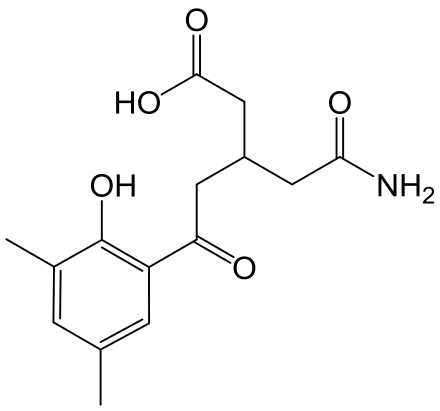

Phenatic acid

Chemical Formula: $C_{15}H_{19}NO_5$

Exact Mass: 293.13

Phenatic acid

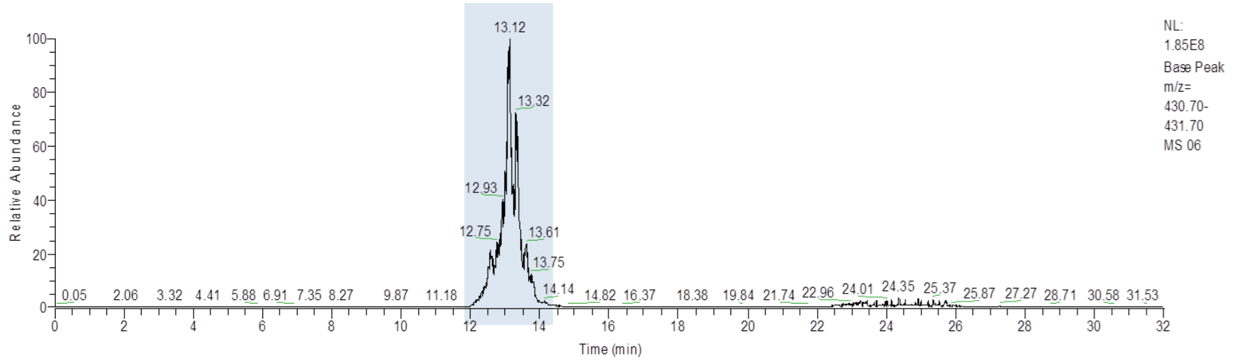

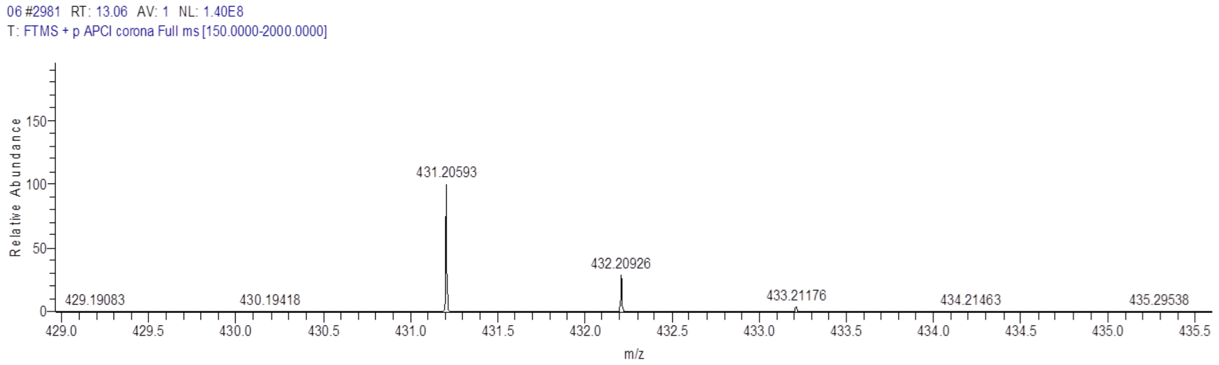

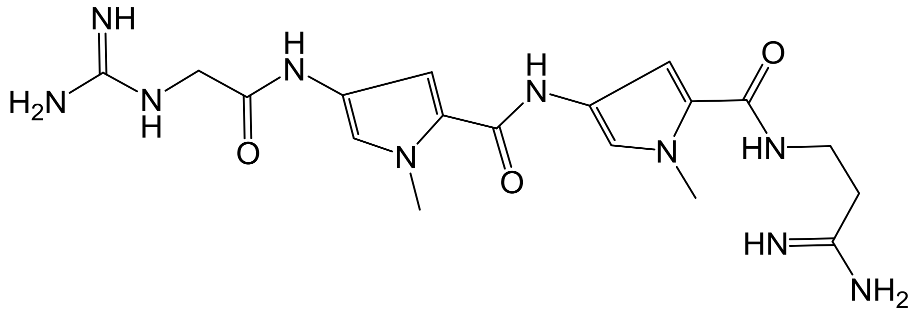

Netropsin

Chemical Formula: $C_{18}H_{26}N_{10}O_{3}$
Exact Mass: 430.22

Netropsin

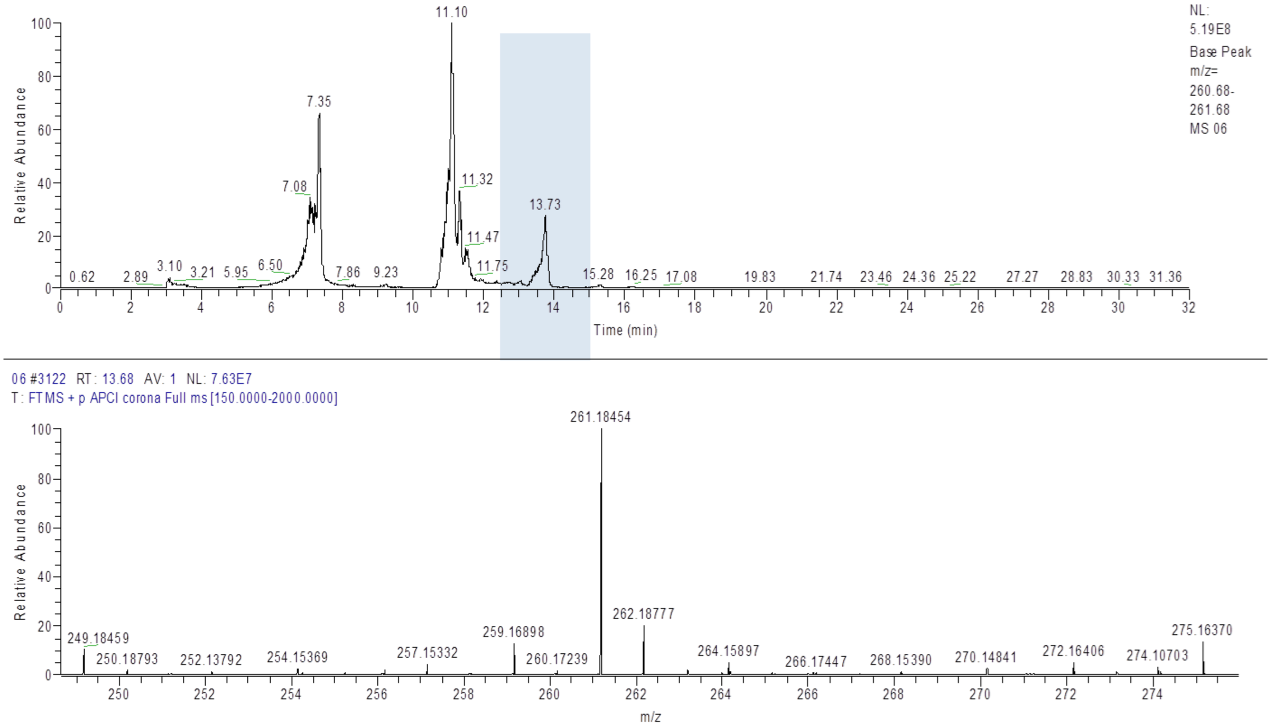

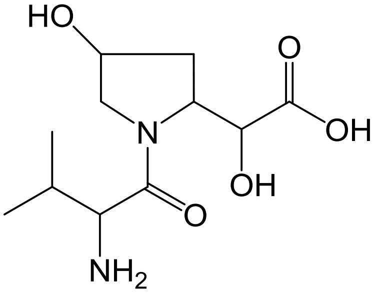

N-Valyldihydroxyhomoproline

Chemical Formula: $C_{11}H_{20}N_2O_5$

Exact Mass: 260.14

N-Valyldihidroxyhomoproline

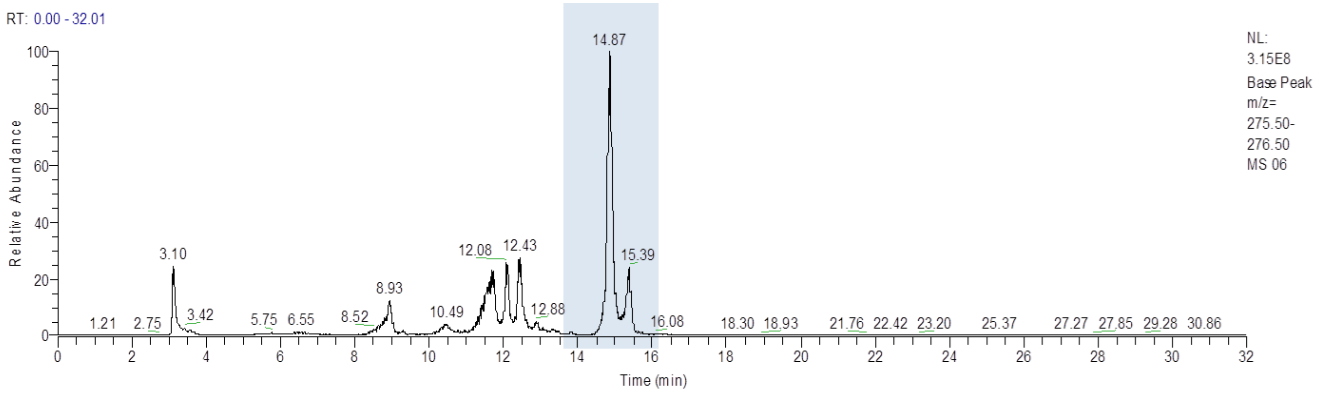

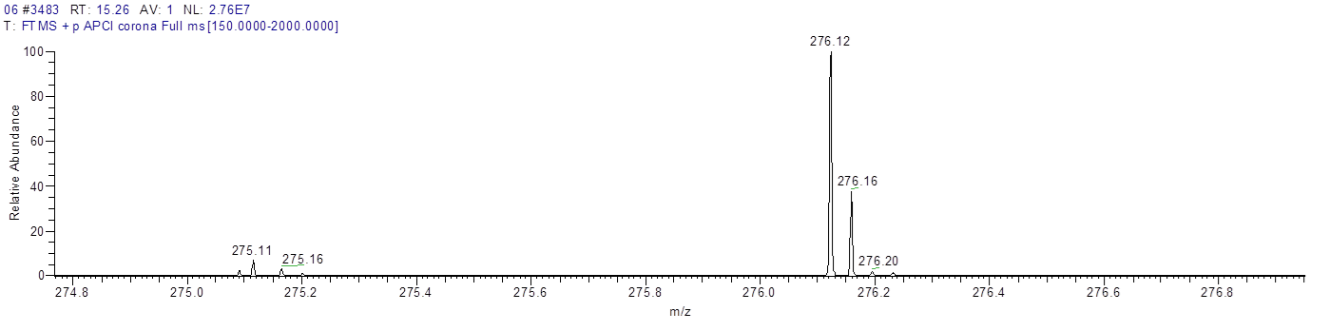

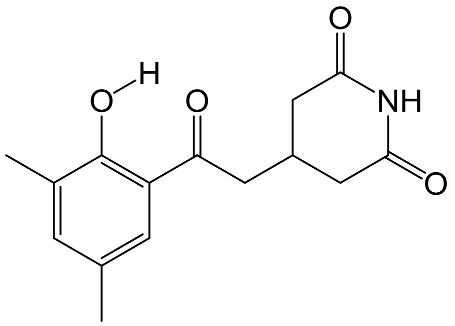

Actiphenol

Chemical Formula: $C_{15}H_{17}NO_4$
Exact Mass: 275.12

Actiphenol

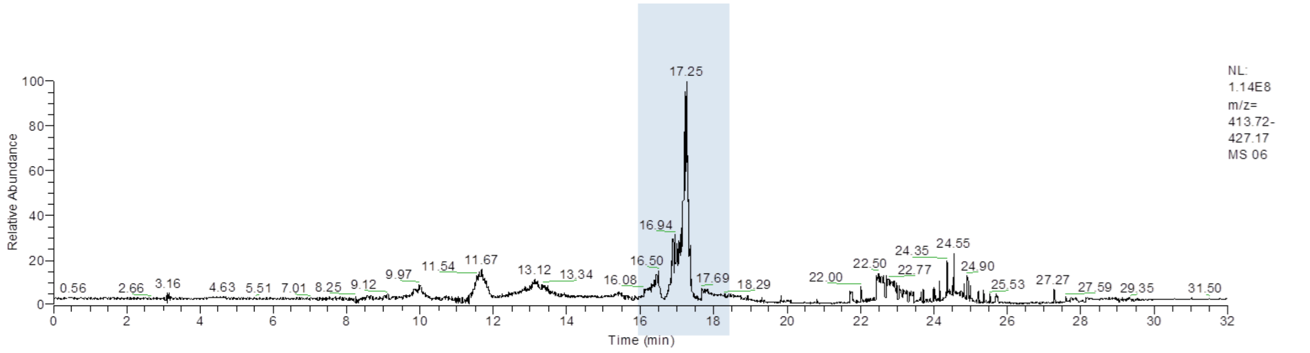

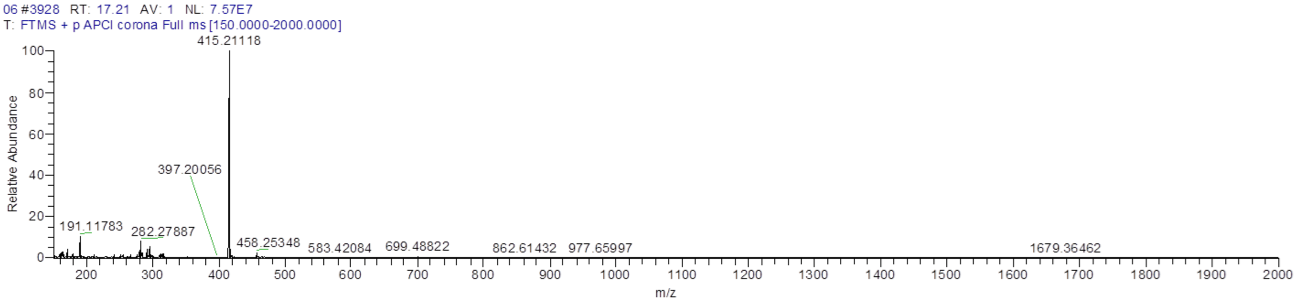

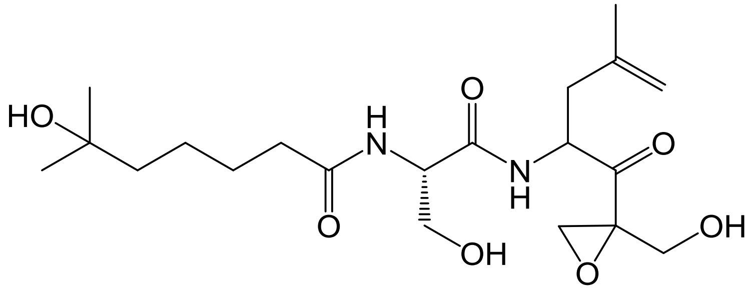

TMC-86B

Chemical Formula: $C_{20}H_{34}N_2O_7$

Exact Mass: 414.24

TMC-86B

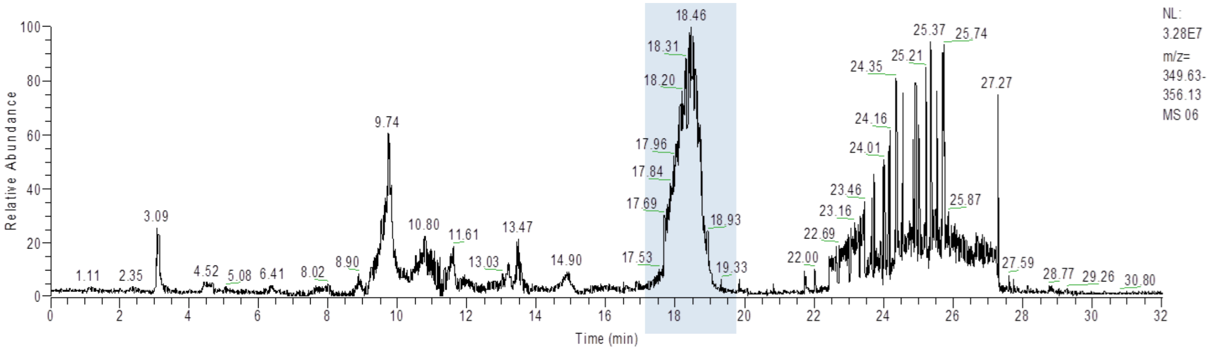

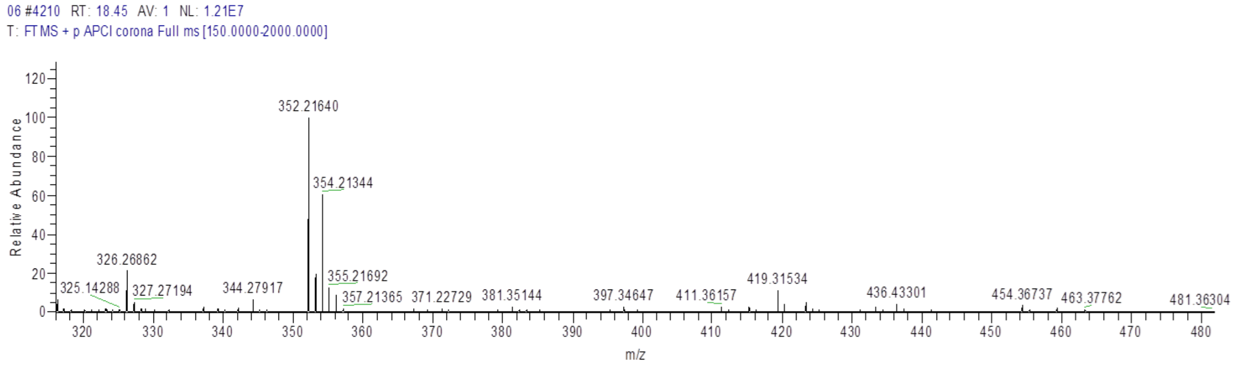

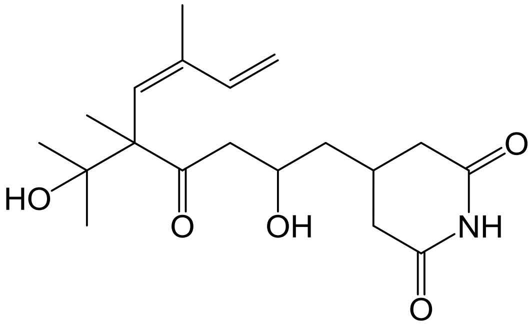

Protomycin

Chemical Formula: $C_{19}H_{29}NO_5$
Exact Mass: 351.20

Protomycin

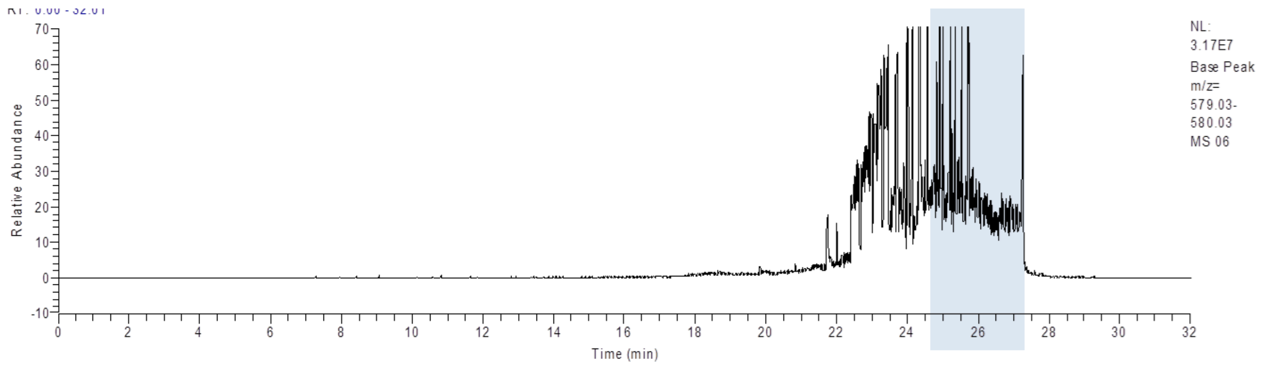

RT: 0.00 - 32.01
NL: 3.17E7
Base Peak m/z= 579.03-580.03
MS 06
Relative Abundance
Time (min)

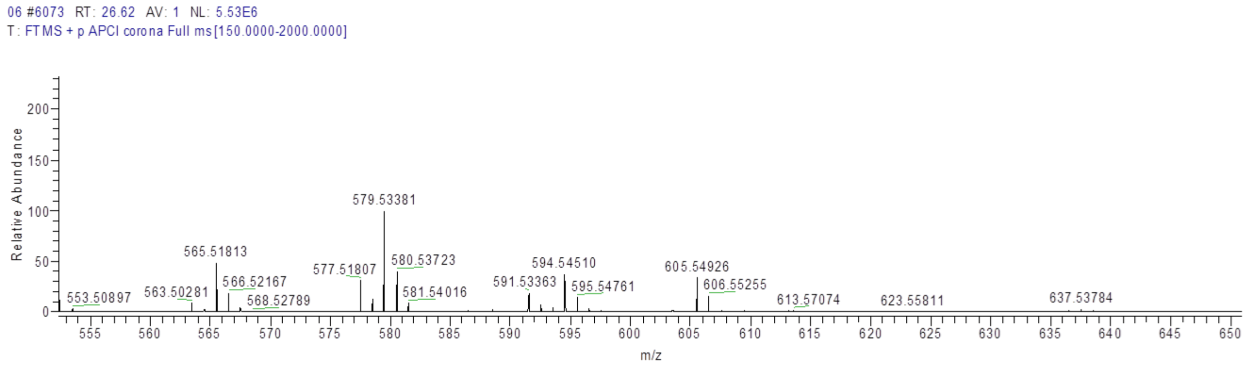

06 #6073 RT: 26.62 AV: 1 NL: 5.53E6
T: FTMS + p APCI corona Full ms [150.0000-2000.0000]
Relative Abundance
579.53381
565.51813
580.53723
594.54510
605.54926
577.51807
566.52167
591.53363
606.55255
595.54761
563.50281
581.54016
553.50897
568.52789
613.57074
623.55811
637.53784
m/z

Unknown

Streptomyces griseus subsp. griseus DSM 40236^T

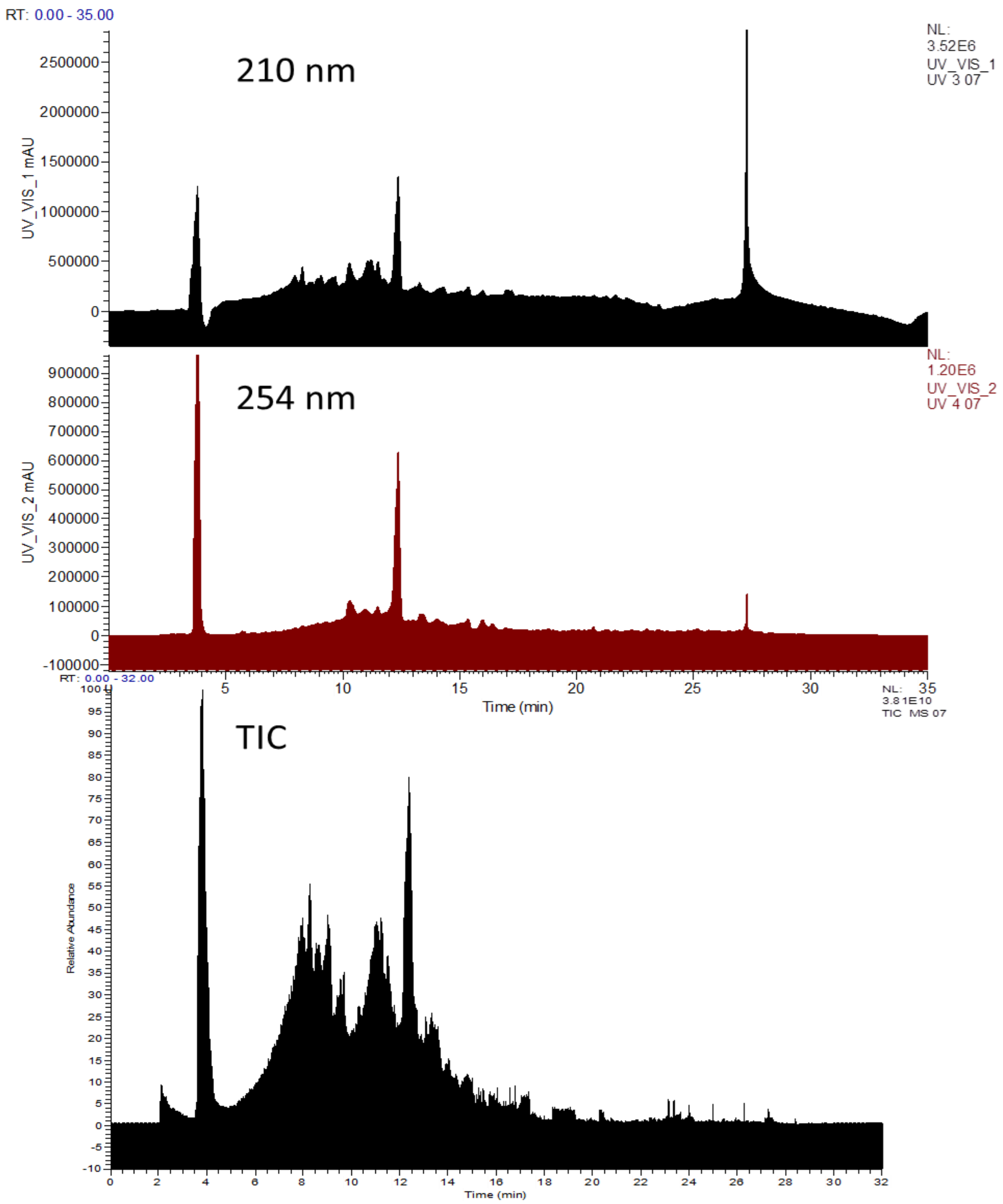

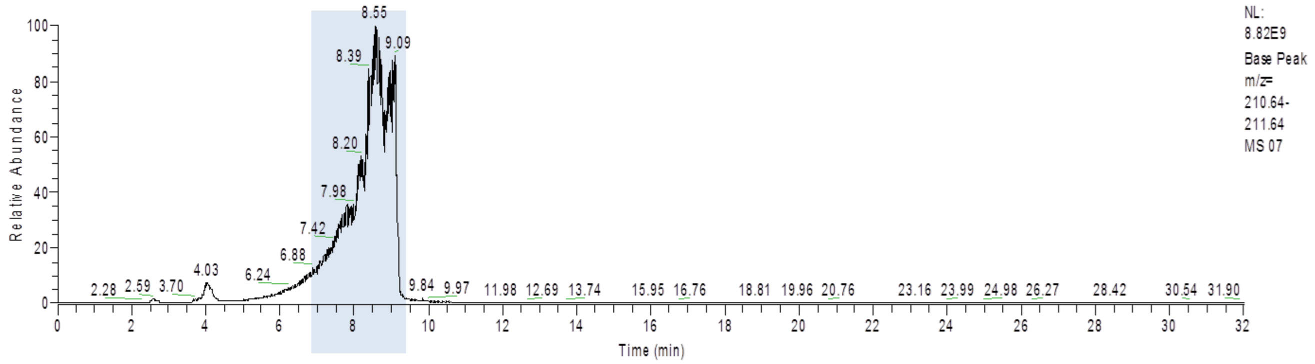

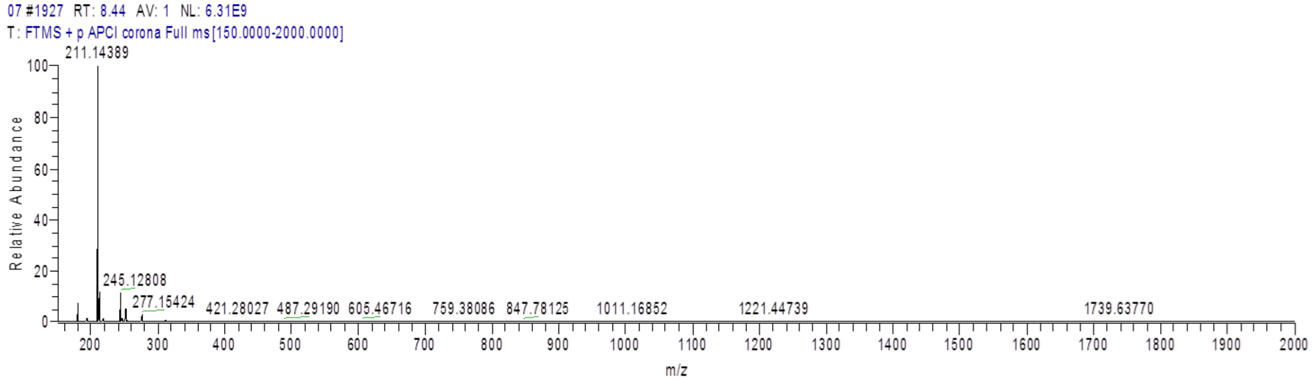

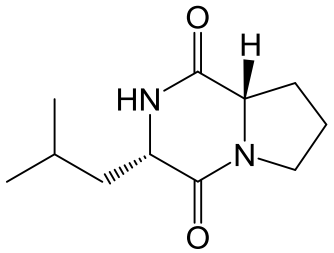

Gancidin W

Chemical Formula: $C_{11}H_{18}N_2O_2$
Exact Mass: 210.14

Gancidin W

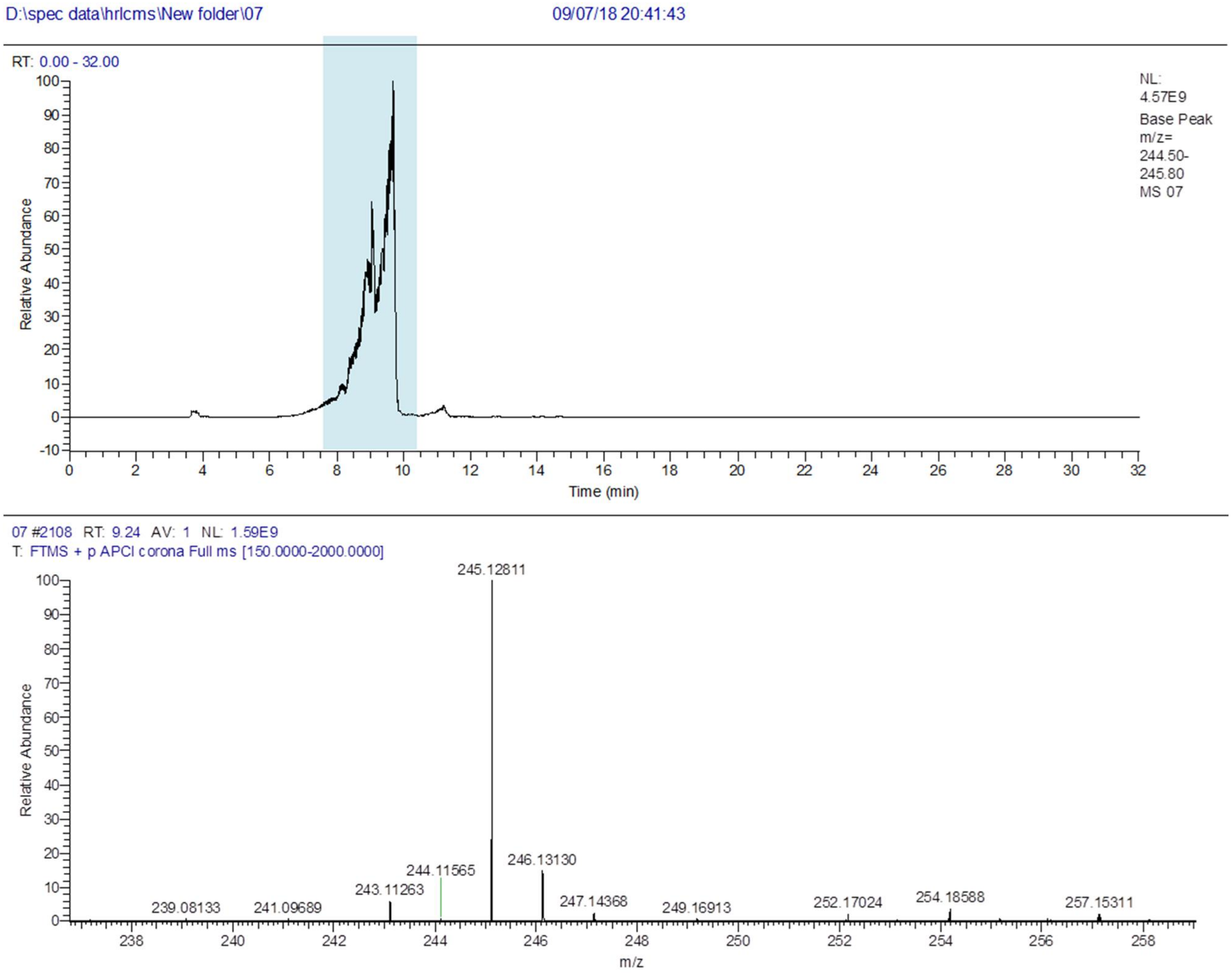

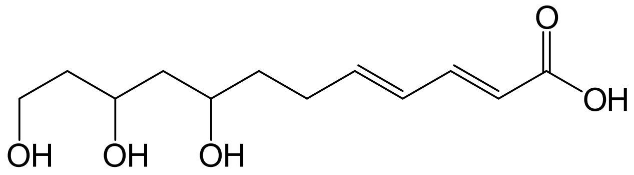

YF-0200-R-B

Exact Mass: 244.13
Molecular Weight: 244.29

YF-0200-R-B

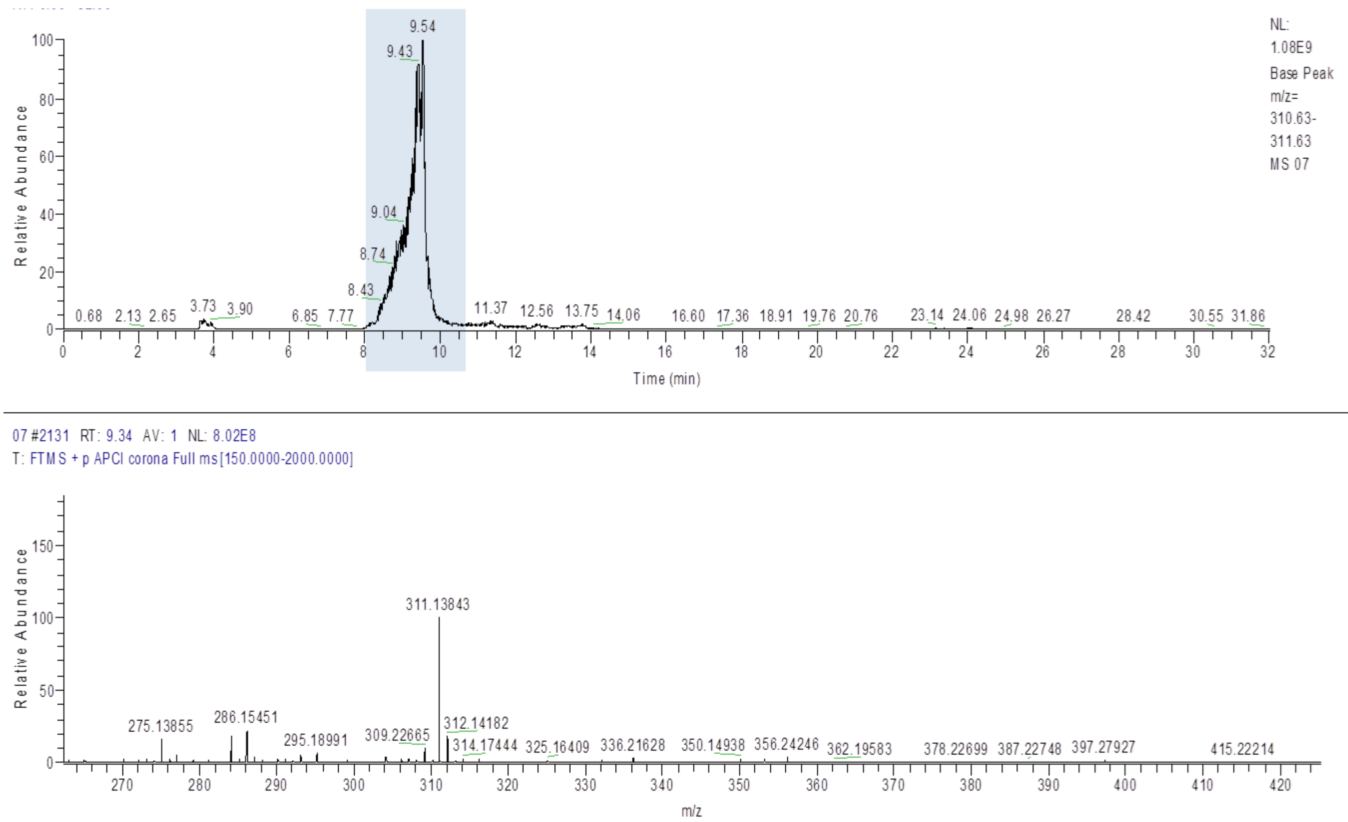

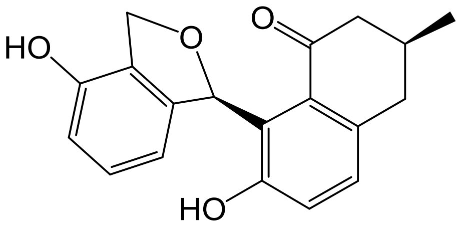

Emycin-E

Chemical Formula: $C_{19}H_{18}O_4$
Exact Mass: 310.12

Emycin E

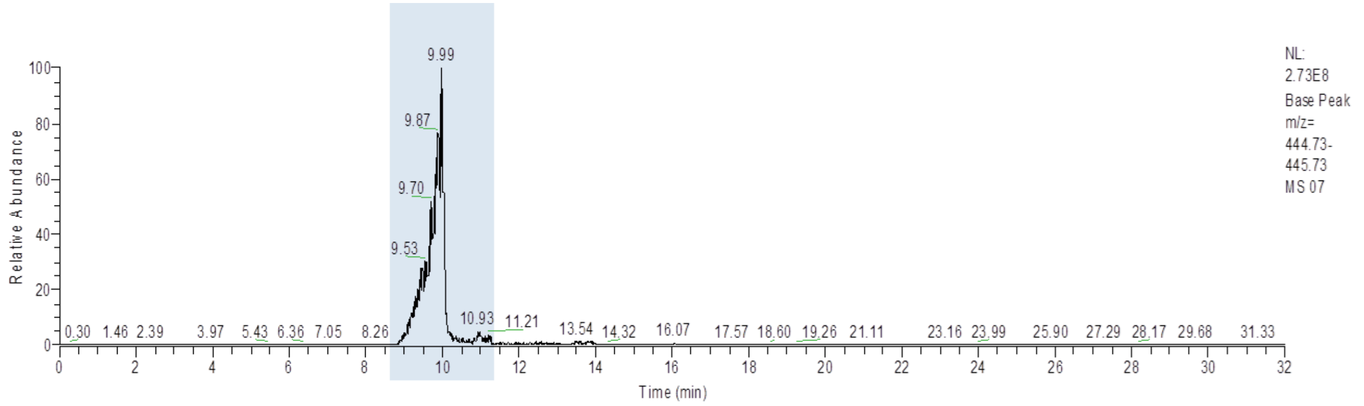

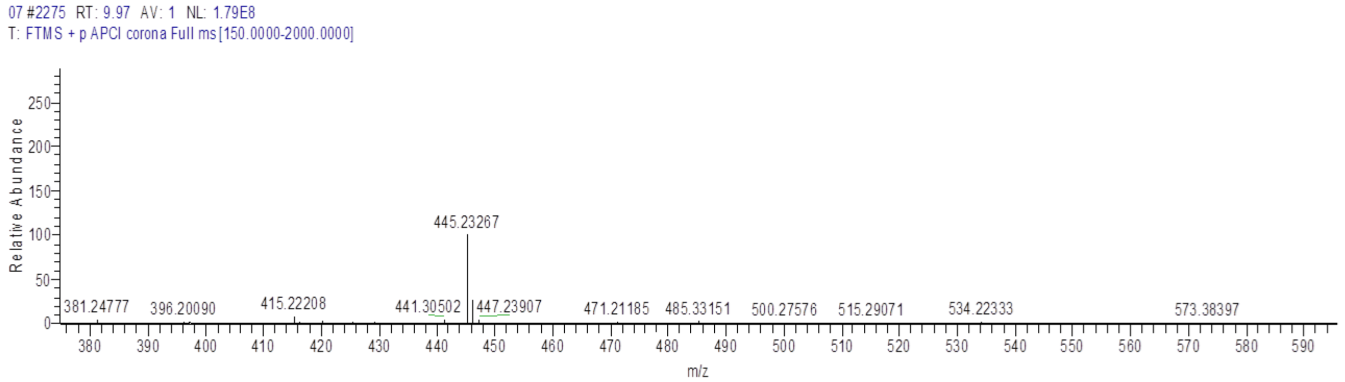

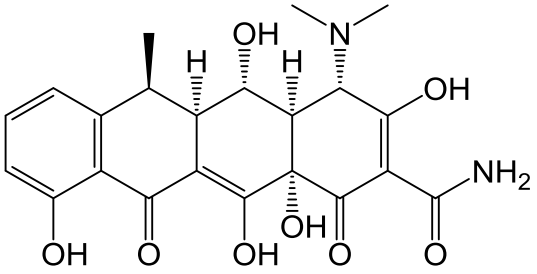

6-beta-deoxy-5-hydroxy-tetracycline

Chemical Formula: $C_{22}H_{24}N_2O_8$
Exact Mass: 444.15

6-beta-deoxy-5-hydroxy-tetracycline

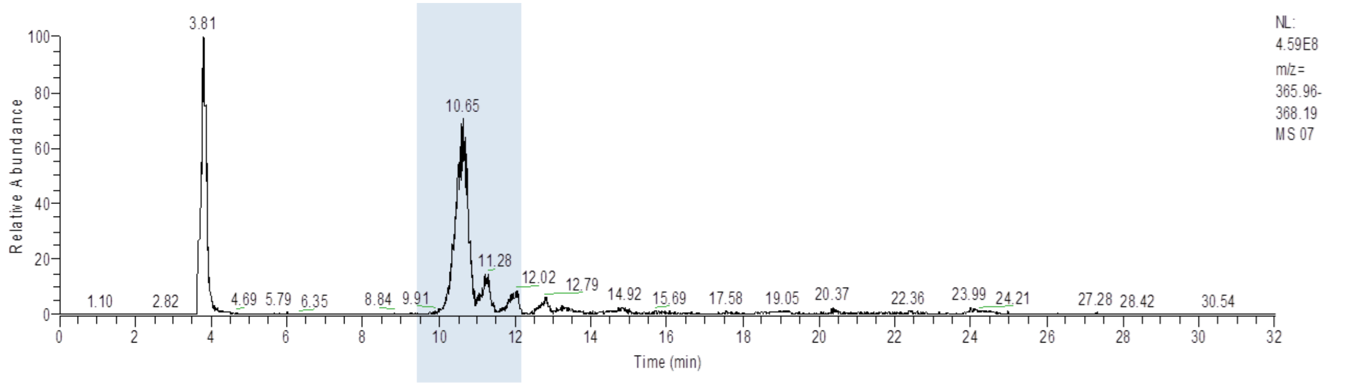

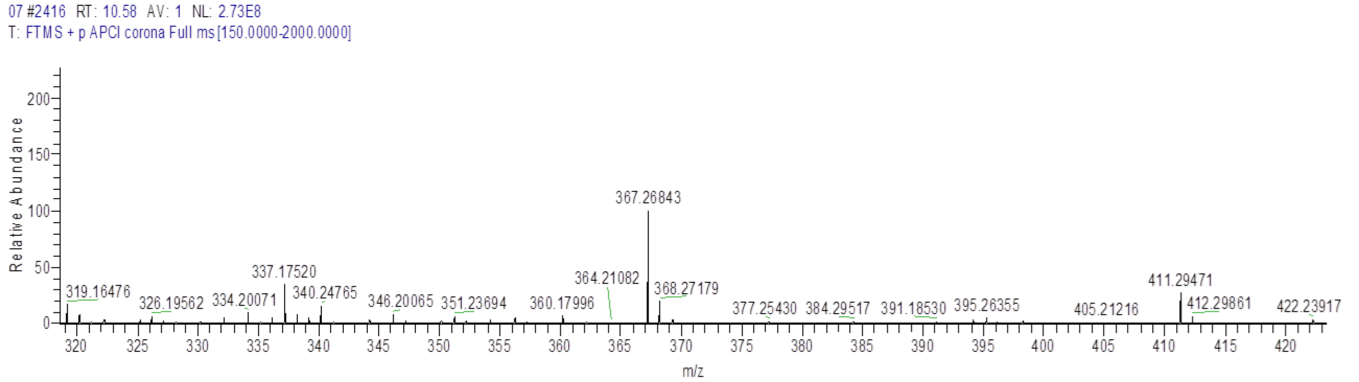

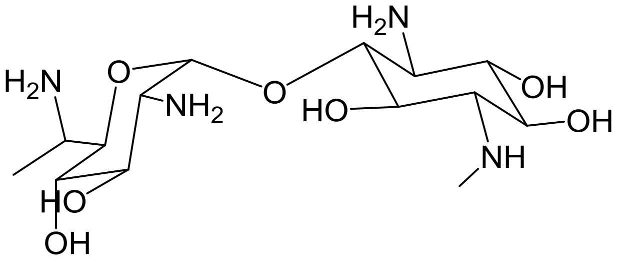

Fortimicin-KK1

Chemical Formula: $C_{14}H_{30}N_4O_7$
Exact Mass: 366.21

Fortimicin KK1

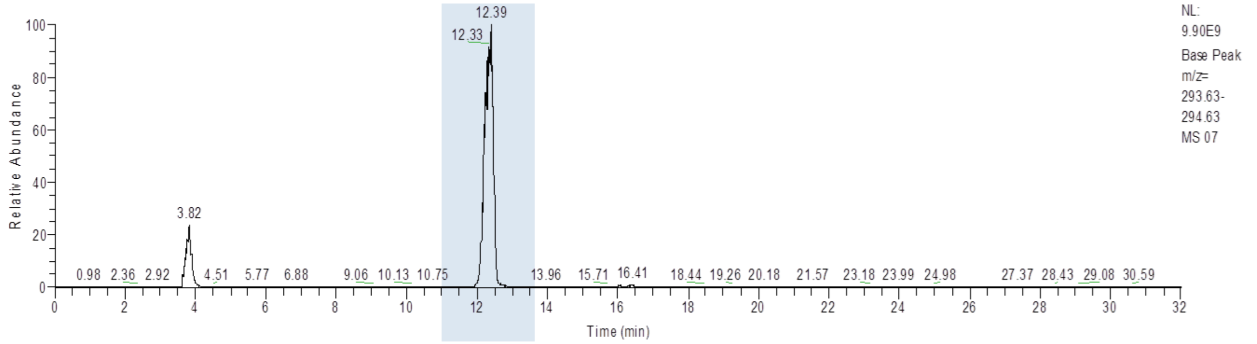

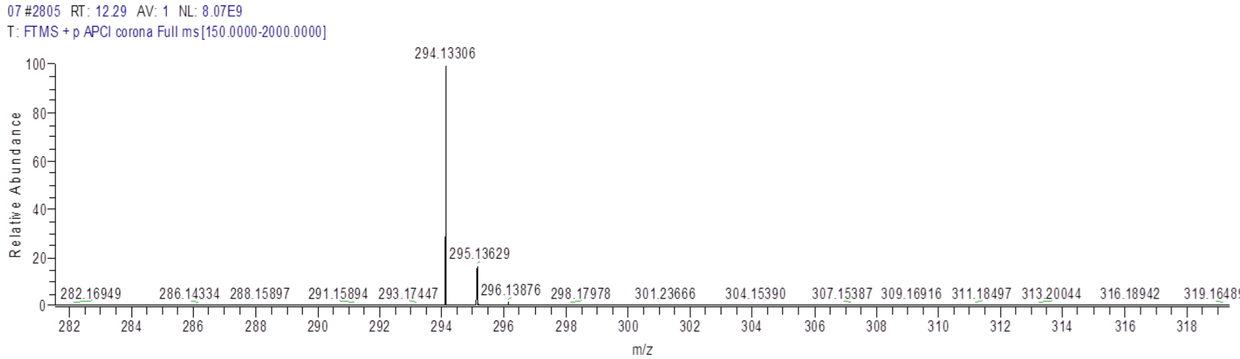

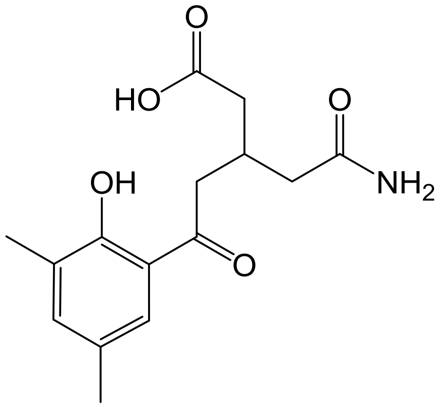

Phenatic acid

Chemical Formula: $C_{15}H_{19}NO_5$

Exact Mass: 293.13

Phenatic acid

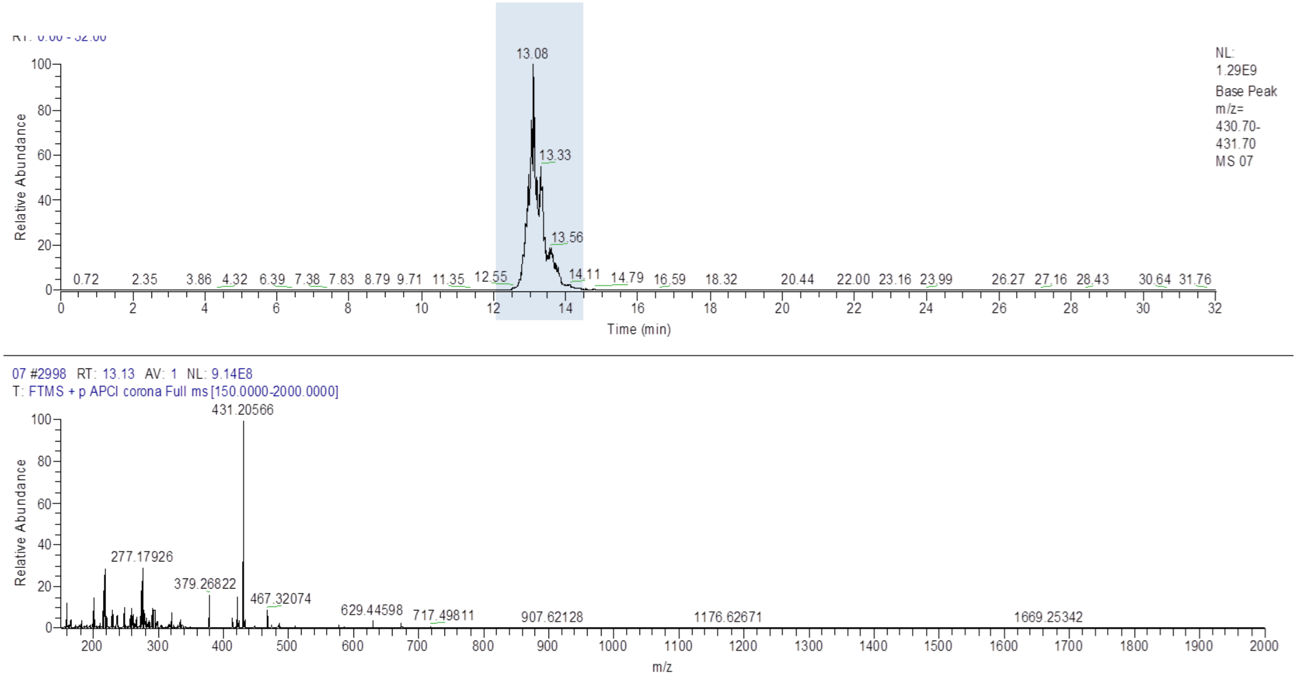

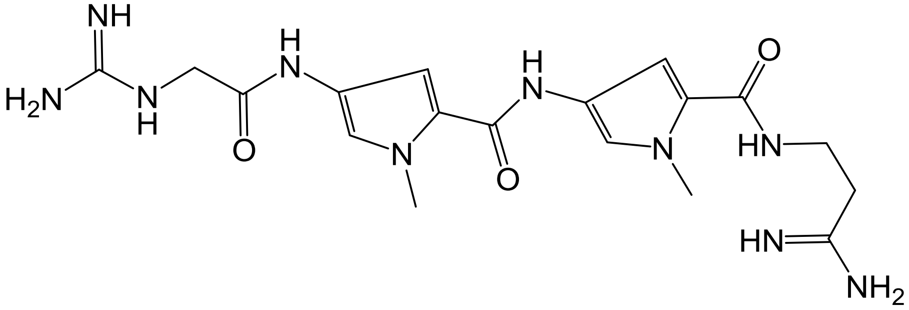

Netropsin

Chemical Formula: $C_{18}H_{26}N_{10}O_3$
Exact Mass: 430.22

Netropsin

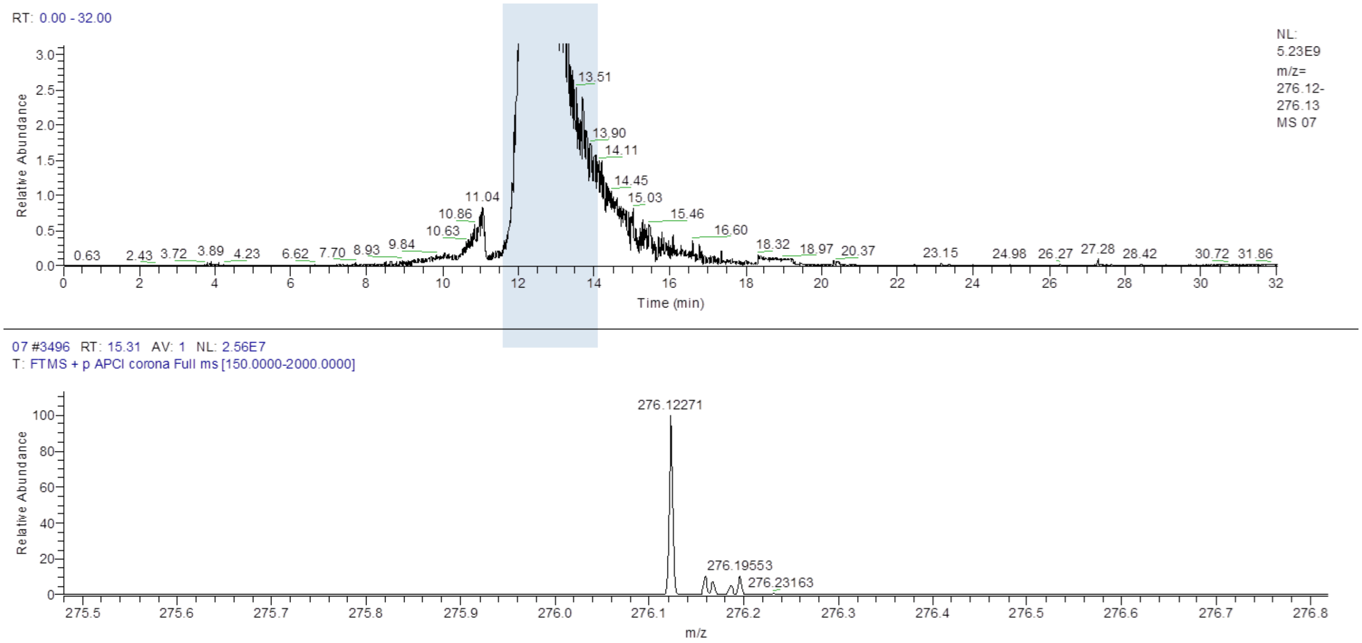

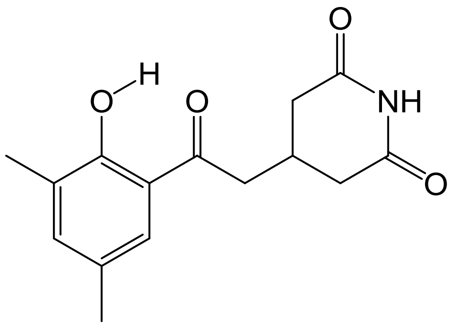

Actiphenol

Chemical Formula: $C_{15}H_{17}NO_4$
Exact Mass: 275.12

Actiphenol

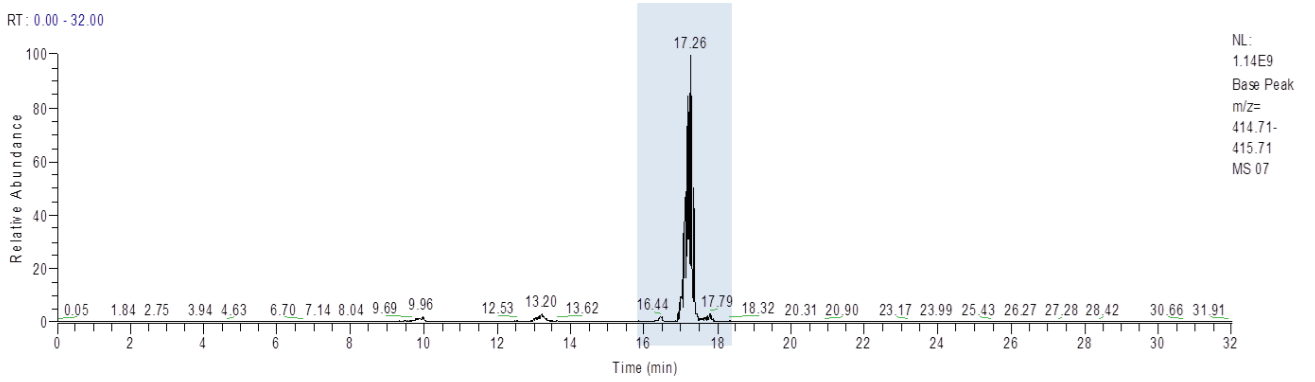

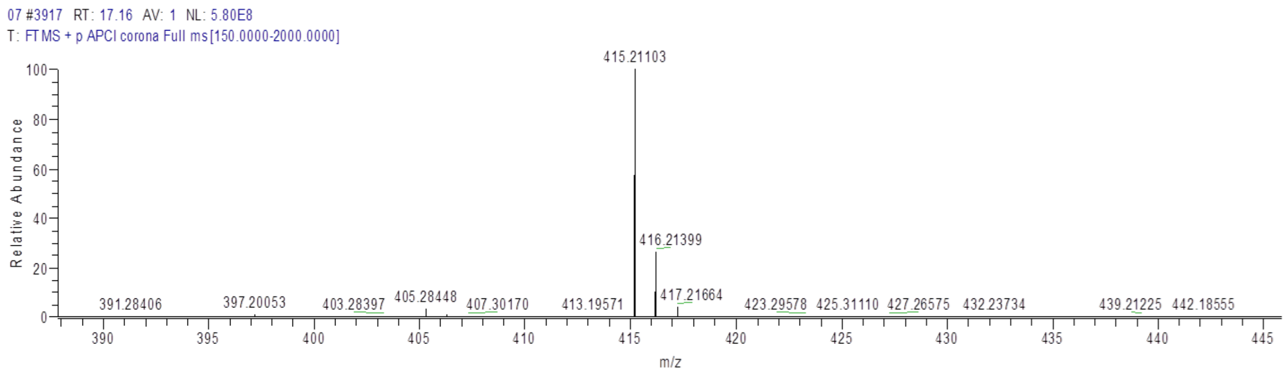

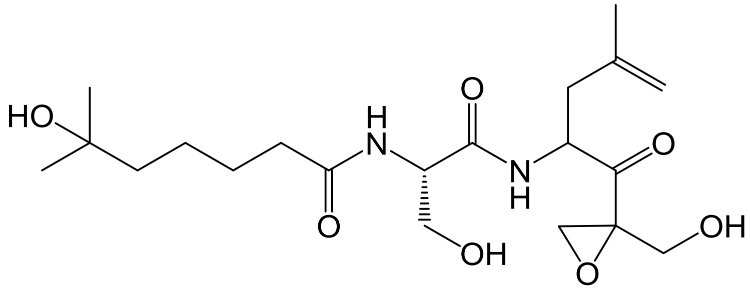

TMC-86B

Chemical Formula: $C_{20}H_{34}N_2O_7$
Exact Mass: 414.24

TMC-86B

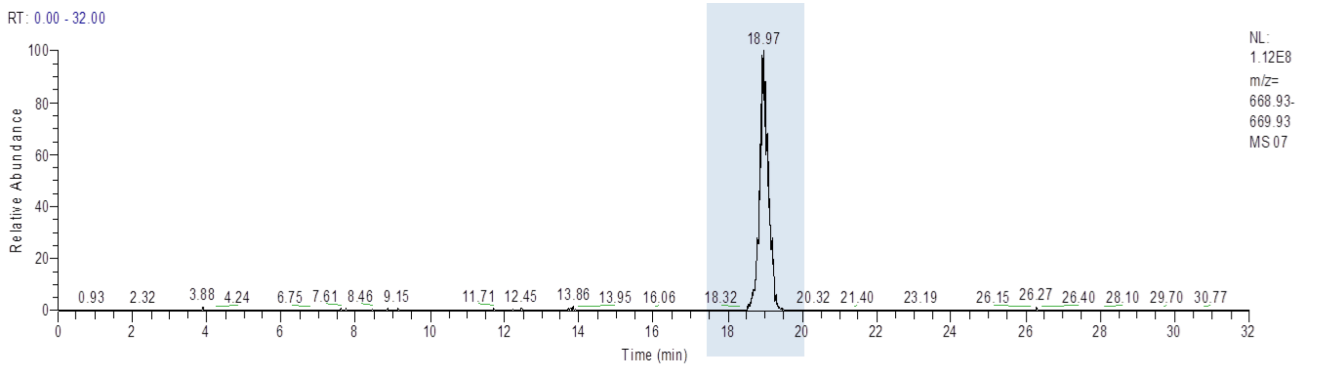

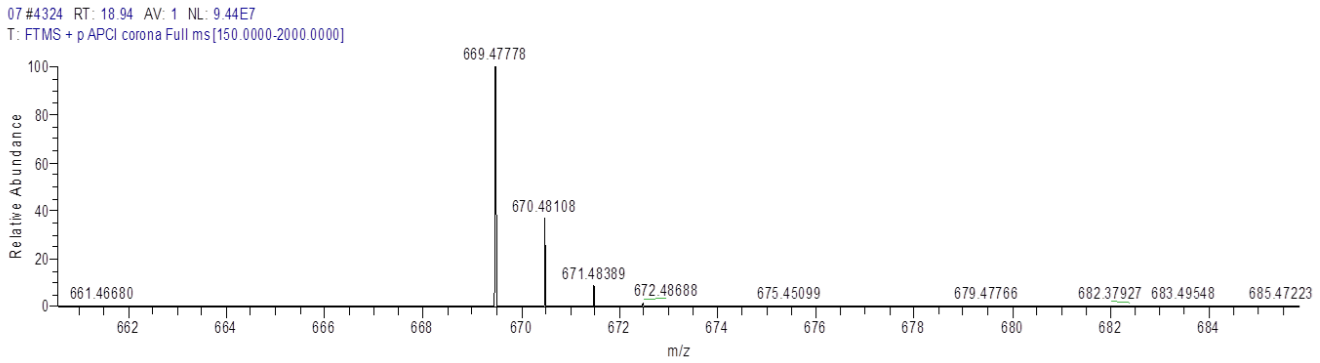

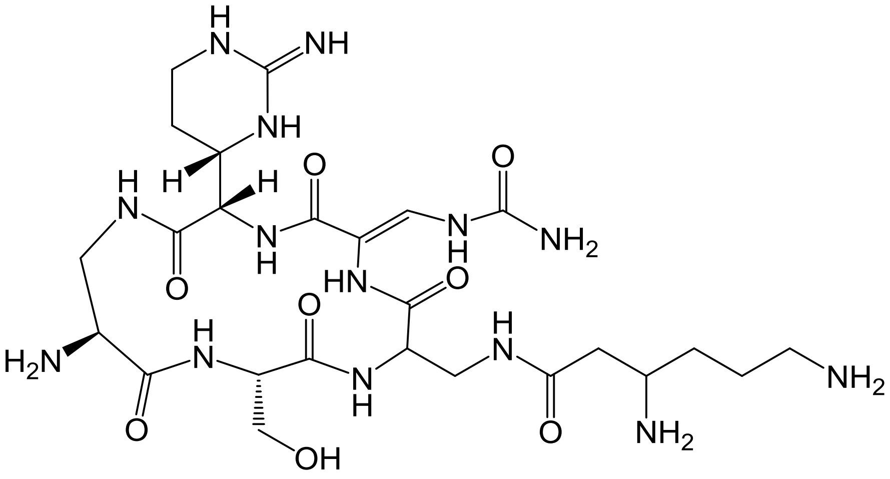

Capromycin

Chemical Formula: $C_{25}H_{44}N_{14}O_8$
Exact Mass: 668.35

Capromycin

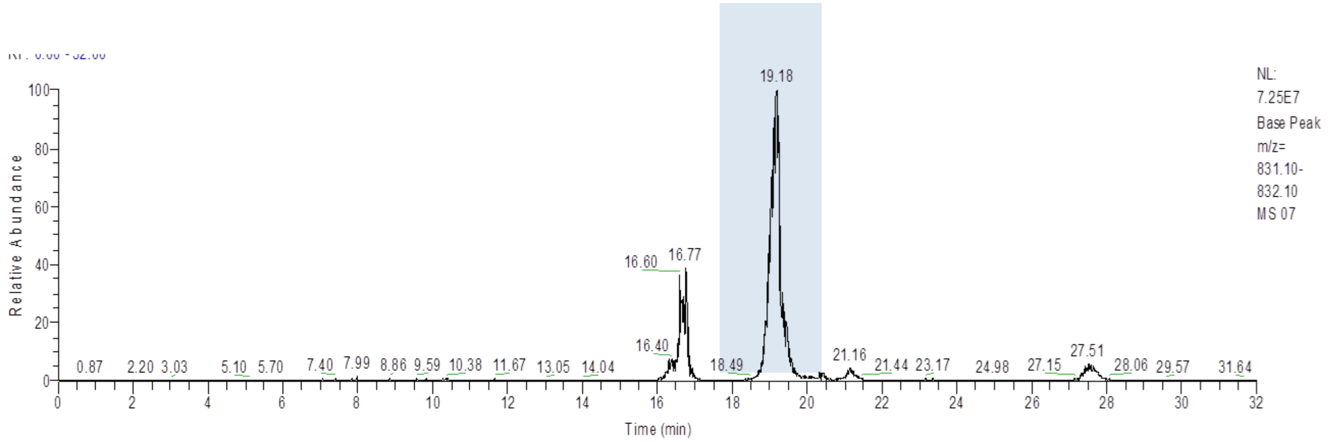

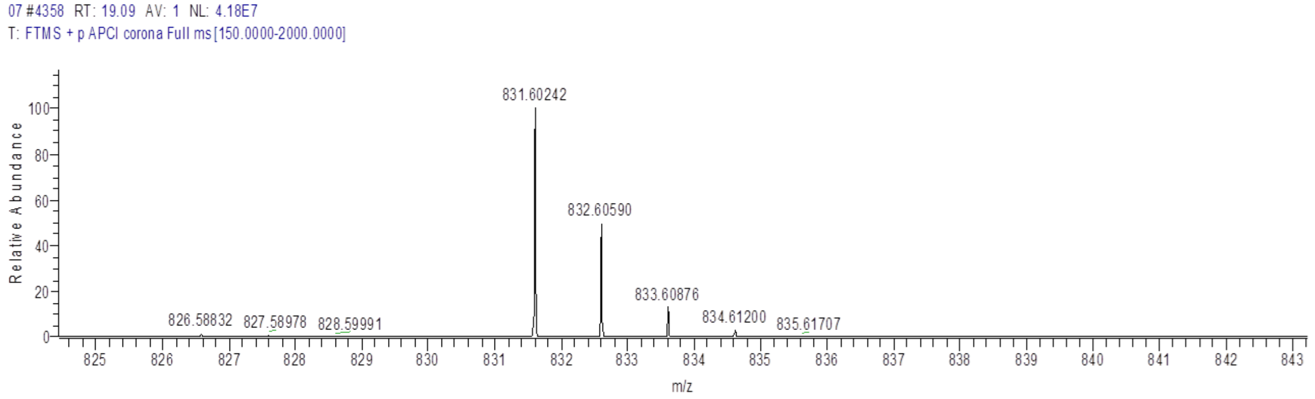

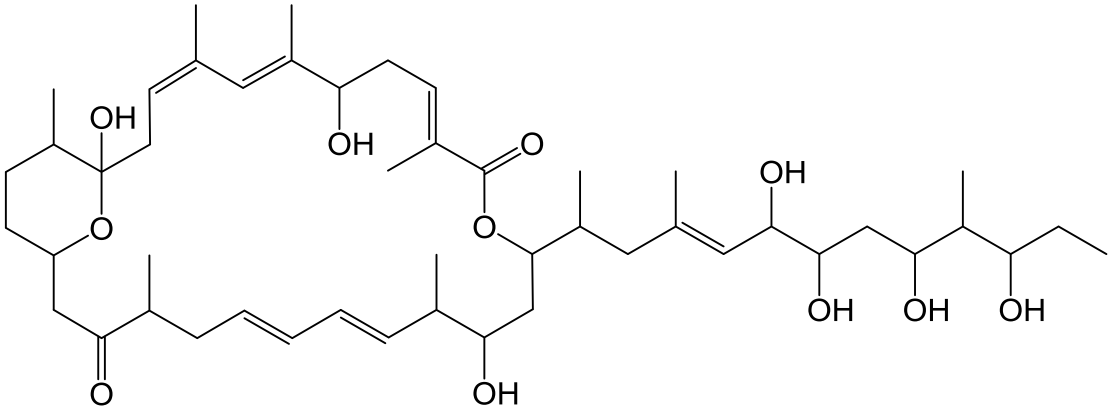

Halstoctacosanolide B

Chemical Formula: $C_{48}H_{78}O_{11}$
Exact Mass: 830.55

Halstoctacosanolide B

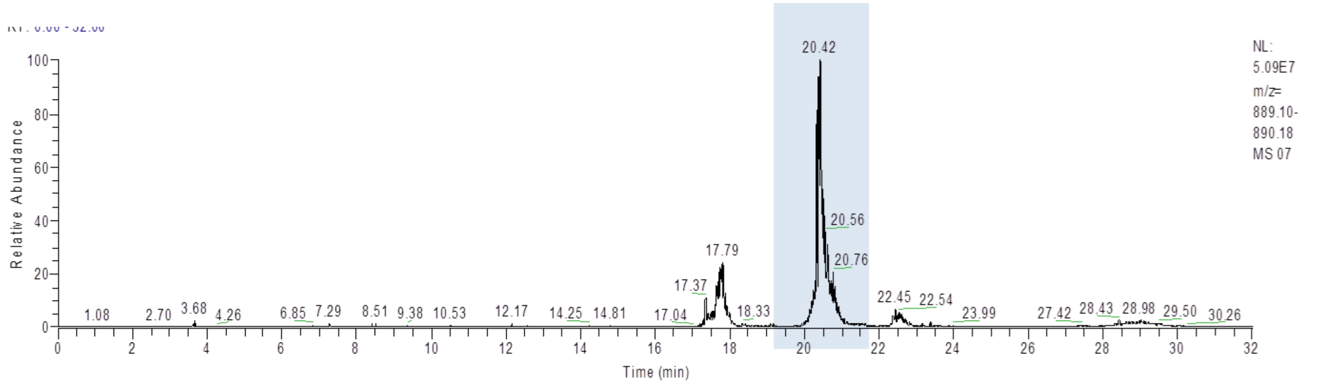

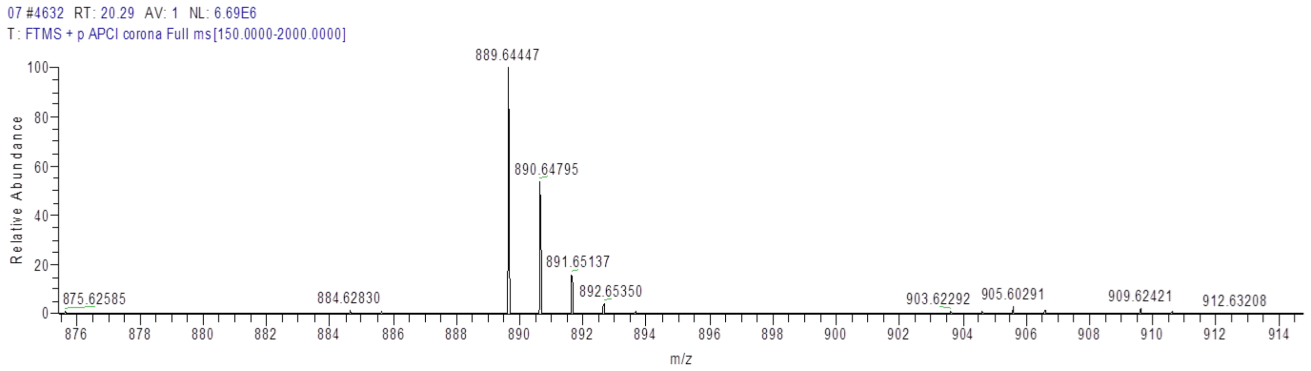

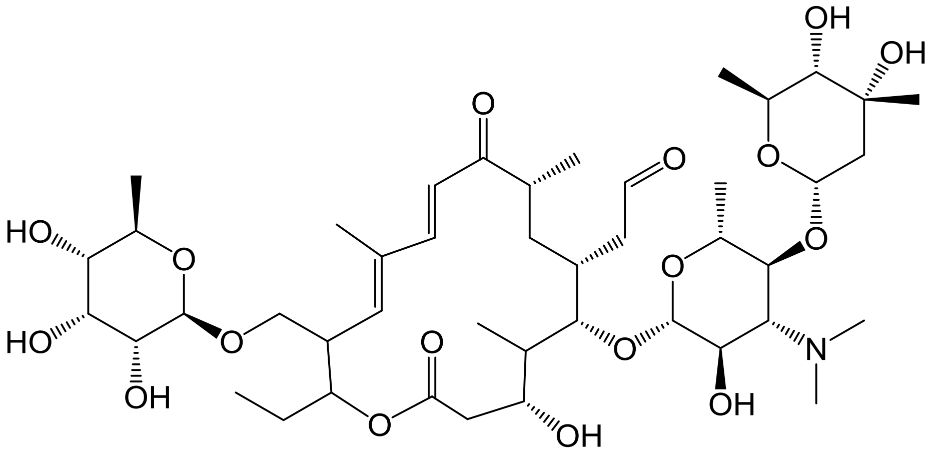

YO - 7625

Chemical Formula: $C_{44}H_{73}NO_{17}$

Exact Mass: 887.49

YO-7625

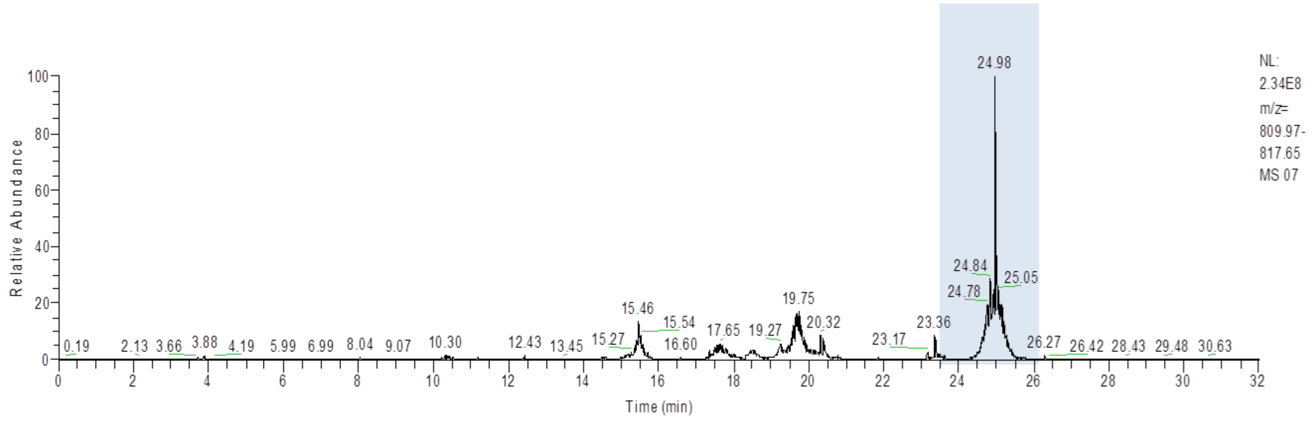
NL: 2.34E8 m/z= 809.97-817.65 MS 07
Relative Abundance
Time (min)
24.98
24.84
25.05
24.78
23.36
19.75
20.32
15.46
15.54
16.60
17.65
19.27
23.17
15.27
26.27
26.42
28.43
29.48
30.63
0.19
2.13
3.66
3.88
4.19
5.99
6.99
8.04
9.07
10.30
12.43
13.45

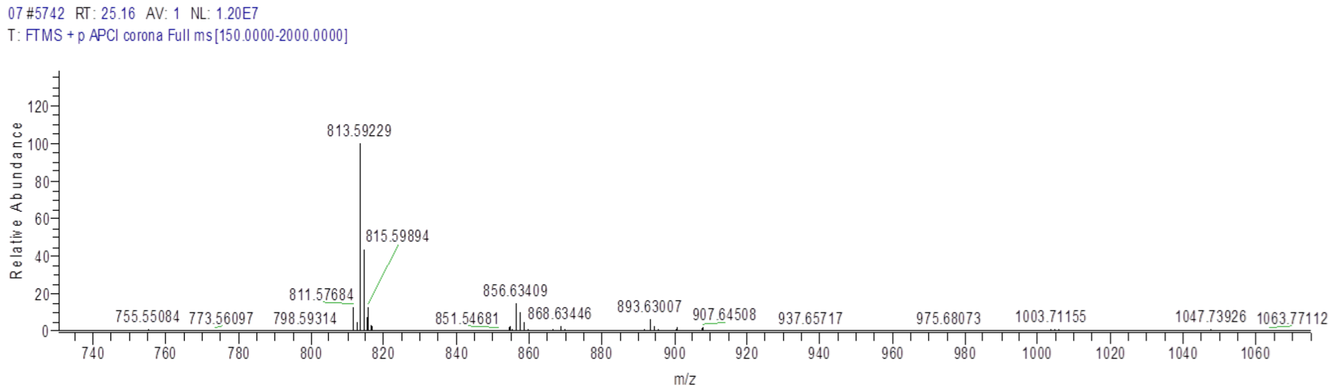
07 #5742 RT: 25.16 AV: 1 NL: 1.20E7
T: FTMS + p APCI corona Full ms [150.0000-2000.0000]
Relative Abundance
m/z
813.59229
815.59894
811.57684
856.63409
868.63446
893.63007
907.64508
937.65717
975.68073
1003.71155
1047.73926
1063.77112
755.55084
773.56097
798.59314
851.54681

Unknown

Streptomyces griseus 16S rRNA comparison

3_1525: *Streptomyces* sp. SN25_8.1 from Easter Island marine sediment.

4_1525: *Streptomyces griseus* subsp. griseus DSM 40236^{T} from a Russian garden soil.

BLAST Results

Questions/comments

Job title: 3_1525

**RID**	TYT6T7XZ01R (Expires on 09-18 16:24 pm)
**Query ID**	lcl|Query_122625
**Description**	3_1525
**Molecule type**	nucleic acid
**Query Length**	1477
**Database Name**	nr
**Description**	Nucleotide collection (nt)
**Program**	BLASTN 2.8.0+

Graphic Summary

Distribution of the top 190 Blast Hits on 100 subject sequences

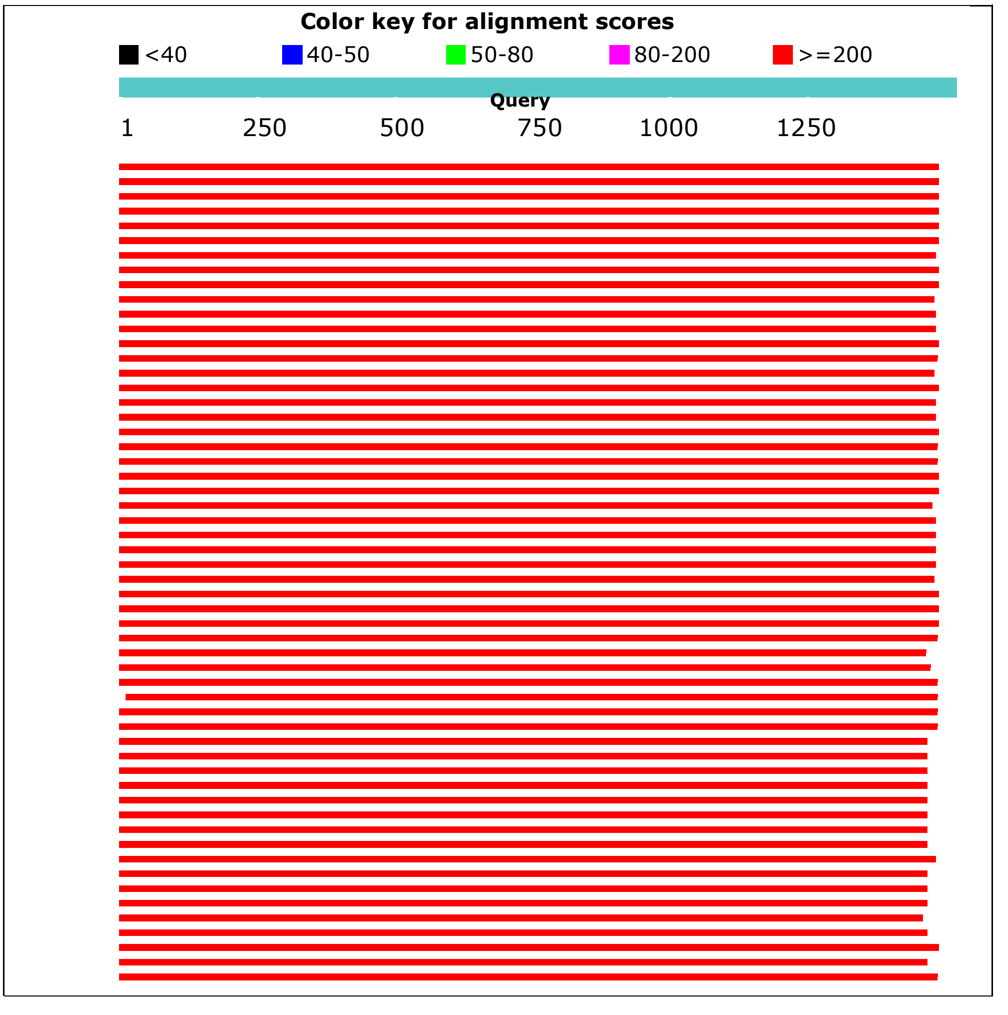

Descriptions

Sequences producing significant alignments:

Description	Max score	Total score	Query cover	E value	Ident	Accession
Streptomyces griseus subsp. griseus NBRC 13350 DNA, complete genome	2721	16327	99%	0.0	99%	AP009493.1
Streptomyces pratensis ATCC 33331, complete genome	2715	16238	99%	0.0	99%	CP002475.1
Streptomyces globisporus strain TFH56 chromosome, complete genome	2710	16238	99%	0.0	99%	CP029361.1
Streptomyces fulvissimus strain DSM 40593 16S ribosomal RNA, partial sequence	2710	2710	99%	0.0	99%	NR_103947.1
Streptomyces fulvissimus DSM 40593, complete genome	2710	16260	99%	0.0	99%	CP005080.1
Streptomyces sp. PAMC26508, complete genome	2710	16257	99%	0.0	99%	CP003990.1
Streptomyces pratensis strain ch24 16S ribosomal RNA, partial sequence	2706	2706	99%	0.0	99%	NR_125621.1
Streptomyces globisporus C-1027, complete genome	2704	16227	99%	0.0	99%	CP013738.1
Streptomyces luridiscabiei strain S63 16S ribosomal RNA, partial sequence	2704	2704	99%	0.0	99%	NR_025155.1
Streptomyces flavolimosus strain CGMCC 2027 16S ribosomal RNA gene, partial sequence	2702	2702	99%	0.0	99%	EF688620.1
Streptomyces praecox strain CGMCC 4.1782 clone 3 16S ribosomal RNA gene, complete sequence	2700	2700	99%	0.0	99%	JQ924404.1
Streptomyces pratensis strain ch24 16S ribosomal RNA, partial sequence	2700	2700	99%	0.0	99%	NR_125619.1
Streptomyces sp. SM18 chromosome, complete genome	2699	16183	99%	0.0	99%	CP029342.1
Streptomyces flavofuscus strain CGMCC 4.1938 clone 3 16S ribosomal RNA gene, complete sequence	2699	2699	99%	0.0	99%	JQ924409.1
Streptomyces griseus strain NRRL B-8030 16S ribosomal RNA gene, partial sequence	2699	2699	99%	0.0	99%	DQ026671.1
Streptomyces sp. Tue6075, complete genome	2695	16157	99%	0.0	99%	CP010833.1
Streptomyces praecox strain CGMCC 4.1782 clone 2 16S ribosomal RNA gene, complete sequence	2695	2695	99%	0.0	99%	JQ924403.1
Streptomyces pratensis strain ch24 16S ribosomal RNA, partial sequence	2695	2695	99%	0.0	99%	NR_125618.1
Streptomyces violaceoruber strain S21, complete genome	2693	16161	99%	0.0	99%	CP020570.1
Streptomyces flavofuscus strain CGMCC 4.1938 clone 2 16S ribosomal RNA gene, complete sequence	2693	2693	99%	0.0	99%	JQ924408.1
Streptomyces flavofuscus strain CGMCC 4.1938 clone 1 16S ribosomal RNA gene, complete sequence	2693	2693	99%	0.0	99%	JQ924407.1
Streptomyces sp. SirexAA-E, complete genome	2693	16133	99%	0.0	99%	CP002993.1
Streptomyces flavofuscus strain NRRL B-2594 16S ribosomal RNA gene, partial sequence	2693	2693	100%	0.0	99%	EF178690.1

Description	Max score	Total score	Query cover	E value	Ident	Accession
Streptomyces microflavus strain NRRL B-2156 16S ribosomal RNA gene, partial sequence	2691	2691	99%	0.0	99%	DQ445795.1
Streptomyces praecox strain CGMCC 4.1782 clone 5 16S ribosomal RNA gene, complete sequence	2689	2689	99%	0.0	99%	JQ924406.1
Streptomyces praecox strain CGMCC 4.1782 clone 4 16S ribosomal RNA gene, complete sequence	2689	2689	99%	0.0	99%	JQ924405.1
Streptomyces praecox strain CGMCC 4.1782 clone 1 16S ribosomal RNA gene, complete sequence	2689	2689	99%	0.0	99%	JQ924402.1
Streptomyces pratensis strain ch24 16S ribosomal RNA, partial sequence	2689	2689	99%	0.0	99%	NR_125620.1
Streptomyces anulatus strain NRRL B-2000 16S ribosomal RNA gene, partial sequence	2689	2689	99%	0.0	99%	DQ026637.1
Streptomyces baarnensis strain NRRL B-1902 16S ribosomal RNA gene, partial sequence	2687	2687	100%	0.0	99%	EF178688.1
Streptomyces flavogriseus partial 16S rRNA gene, type strain CBS 101.34T	2687	2687	99%	0.0	99%	AJ494864.1
Streptomyces sp. CFMR 7 strain CFMR-7, complete genome	2682	15924	99%	0.0	99%	CP011522.1
Streptomyces flavovirens strain CGMCC 4.575 clone 1 16S ribosomal RNA gene, complete sequence	2682	2682	99%	0.0	99%	JQ924386.1
Streptomyces griseus subsp. griseus gene for 16S rRNA, partial sequence, strain: NBRC 15744	2680	2680	98%	0.0	99%	AB184699.1
Streptomyces argenteolus strain JCM 4623 16S ribosomal RNA, partial sequence	2680	2680	99%	0.0	99%	NR_112120.1
Streptomyces flavovirens strain CGMCC 4.575 clone 2 16S ribosomal RNA gene, complete sequence	2678	2678	99%	0.0	99%	JQ924387.1
Streptomyces sp. YM5-799 gene for 16S rRNA, partial sequence	2678	2678	98%	0.0	99%	AB534176.1
Streptomyces flavovirens strain CGMCC 4.575 clone 5 16S ribosomal RNA gene, complete sequence	2676	2676	99%	0.0	99%	JQ924389.1
Streptomyces flavovirens strain CGMCC 4.575 clone 4 16S ribosomal RNA gene, complete sequence	2676	2676	99%	0.0	99%	JQ924388.1
Streptomyces microflavus strain NRRL B-1332 16S ribosomal RNA gene, partial sequence	2675	2675	98%	0.0	99%	EF178673.1
Streptomyces paresii 16S rRNA gene, type strain LMG 23704T	2675	2675	98%	0.0	99%	AJ969177.1
Streptomyces anulatus gene for 16S rRNA, partial sequence, strain: NBRC 13369	2673	2673	98%	0.0	99%	AB184875.1
Streptomyces cavourensis subsp. washingtonensis gene for 16S rRNA, partial sequence, strain: NBRC 15391	2673	2673	98%	0.0	99%	AB184642.1
Streptomyces acidoresistans gene for 16S rRNA, partial sequence, strain: NBRC 13442	2673	2673	98%	0.0	99%	AB184406.1
Streptomyces praecox gene for 16S rRNA, partial sequence, strain: NBRC 13073	2673	2673	98%	0.0	99%	AB184293.1
Streptomyces alboviridis gene for 16S rRNA, partial sequence, strain: NBRC 13013	2673	2673	98%	0.0	99%	AB184256.1

Description	Max score	Total score	Query cover	E value	Ident	Accession
Streptomyces argenteolus gene for 16S rRNA, partial sequence, strain: NBRC 12841	2673	2673	98%	0.0	99%	AB184187.1
Streptomyces finlayi strain CGMCC 4.1436 clone 1 16S ribosomal RNA gene, complete sequence	2669	2669	99%	0.0	99%	JQ924390.1
Streptomyces rubiginosohelvolus gene for 16S rRNA, partial sequence, strain: NBRC 12912	2669	2669	98%	0.0	99%	AB184240.2
Streptomyces microflavus gene for 16S rRNA, partial sequence, strain: NBRC 13062	2669	2669	98%	0.0	99%	AB184284.1
Streptomyces fulvorobeus gene for 16S rRNA, partial sequence, strain: NBRC 15897	2667	2667	98%	0.0	99%	AB184711.1
Streptomyces griseus subsp. griseus gene for 16S rRNA, partial sequence, strain: NBRC 12875	2667	2667	97%	0.0	99%	AB184211.1
Streptomyces griseinus gene for 16S rRNA, partial sequence, strain: NBRC 12869	2667	2667	98%	0.0	99%	AB184205.1
Streptomyces sp. S8, complete genome	2665	15950	99%	0.0	99%	CP015362.1
Streptomyces flavofuscus gene for 16S rRNA, partial sequence, strain: NBRC 100768	2665	2665	98%	0.0	99%	AB249935.1
Streptomyces clavifer strain NRRL B-2557 16S ribosomal RNA gene, partial sequence	2663	2663	99%	0.0	99%	DQ026670.1
Streptomyces albovinaceus gene for 16S rRNA, partial sequence, strain: NBRC 12739	2663	2663	98%	0.0	99%	AB249958.1
Streptomyces erumpens gene for 16S rRNA, partial sequence, strain: NBRC 15403	2663	2663	97%	0.0	99%	AB184654.1
Streptomyces baarnensis gene for 16S rRNA, partial sequence, strain: NBRC 14727	2663	2663	98%	0.0	99%	AB184615.1
Streptomyces fimicarius gene for 16S rRNA, partial sequence, strain: NBRC 13037	2663	2663	98%	0.0	99%	AB184269.1
Streptomyces globisporus subsp. globisporus gene for 16S rRNA, partial sequence, strain: NBRC 12867	2663	2663	98%	0.0	99%	AB184203.1
Streptomyces cavourensis strain TJ430 chromosome, complete genome	2662	15856	99%	0.0	99%	CP030930.1
Streptomyces bacillaris strain ATCC 15855 chromosome, complete genome	2662	15972	99%	0.0	99%	CP029378.1
Streptomyces cavourensis strain 1AS2a chromosome, complete genome	2662	15928	99%	0.0	99%	CP024957.1
Streptomyces globisporus subsp. globisporus strain NRRL B-2872 16S ribosomal RNA gene, partial sequence	2662	2662	98%	0.0	99%	EF178686.1
Streptomyces griseolus gene for 16S rRNA, partial sequence, strain: NBRC 3415	2662	2662	98%	0.0	99%	AB184768.1
Streptomyces puniceus gene for 16S rRNA, partial sequence, strain: NBRC 12811	2662	2662	98%	0.0	99%	AB184163.1
Streptomyces halstedii gene for 16S rRNA, partial sequence, strain: NBRC 12783	2662	2662	98%	0.0	99%	AB184142.1

Description	Max score	Total score	Query cover	E value	Ident	Accession
Streptomyces atratus strain SCSIO_ZH16 chromosome, complete genome	2660	15961	99%	0.0	99%	CP027306.1
Streptomyces flavovirens strain NRRL B-2685 16S ribosomal RNA gene, partial sequence	2660	2660	99%	0.0	99%	DQ026635.1
Streptomyces pluricolorescens gene for 16S rRNA, partial sequence, strain: NBRC 12808	2660	2660	98%	0.0	99%	AB184162.1
Streptomyces olivoviridis strain CGMCC 4.1739 clone 2 16S ribosomal RNA gene, complete sequence	2658	2658	99%	0.0	99%	JQ924394.1
Streptomyces finlayi strain CGMCC 4.1436 clone 2 16S ribosomal RNA gene, complete sequence	2658	2658	99%	0.0	99%	JQ924391.1
Streptomyces californicus gene for 16S rRNA, partial sequence, strain: NBRC 12750	2658	2658	98%	0.0	99%	AB184116.2
Streptomyces cinereorectus gene for 16S rRNA, partial sequence, strain: NBRC 15395	2658	2658	98%	0.0	99%	AB184646.1
Streptomyces mutomycini strain CGMCC 4.1747 clone 3 16S ribosomal RNA gene, complete sequence	2656	2656	99%	0.0	99%	JQ924399.1
Streptomyces halstedii strain NRRL B-1238 16S ribosomal RNA gene, partial sequence	2656	2656	98%	0.0	99%	EF178695.1
Streptomyces parvus gene for 16S rRNA, partial sequence, strain: NBRC 3388	2656	2656	98%	0.0	99%	AB184756.1
Streptomyces californicus gene for 16S rRNA, partial sequence, strain: NBRC 3386	2656	2656	98%	0.0	99%	AB184755.1
Streptomyces badius gene for 16S rRNA, partial sequence, strain: NBRC 12745	2656	2656	98%	0.0	99%	AB184114.1
Streptomyces lunaelactis strain MM109 chromosome, complete genome	2654	15660	99%	0.0	99%	CP026304.1
Streptomyces nitrosporeus strain CGMCC 4.1973 clone 4 16S ribosomal RNA gene, complete sequence	2654	2654	99%	0.0	99%	JQ924413.1
Streptomyces anulatus strain NRRL B-2873 16S ribosomal RNA gene, partial sequence	2654	2654	97%	0.0	99%	DQ026639.1
Streptomyces sindenensis gene for 16S rRNA, partial sequence, strain: NBRC 3399	2654	2654	98%	0.0	99%	AB184759.1
Streptomyces mediolani gene for 16S rRNA, partial sequence, strain: NBRC 15427	2654	2654	98%	0.0	99%	AB184674.1
Streptomyces olivoviridis strain CGMCC 4.1739 clone 5 16S ribosomal RNA gene, complete sequence	2652	2652	99%	0.0	99%	JQ924396.1
Streptomyces olivoviridis strain CGMCC 4.1739 clone 4 16S ribosomal RNA gene, complete sequence	2652	2652	99%	0.0	99%	JQ924395.1
Kitasatospora albolonga strain YIM 101047, complete genome	2651	15858	99%	0.0	99%	CP020563.1
Streptomyces mutomycini strain CGMCC 4.1747 clone 1 16S ribosomal RNA gene, complete sequence	2651	2651	99%	0.0	99%	JQ924397.1

Description	Max score	Total score	Query cover	E value	Ident	Accession
Streptomyces flavovirens gene for 16S rRNA, partial sequence, strain: NBRC 3197	2651	2651	98%	0.0	99%	AB184827.1
Streptomyces ornatus gene for 16S rRNA, partial sequence, strain: NBRC 13069	2651	2651	97%	0.0	99%	AB184290.1
Streptomyces flavogriseus gene for 16S rRNA, partial sequence, strain: NBRC 13040	2651	2651	98%	0.0	99%	AB184271.1
Streptomyces flavovirens gene for 16S rRNA, partial sequence, strain: NBRC 12771	2651	2651	98%	0.0	99%	AB184133.1
Streptomyces nitrosporeus strain CGMCC 4.1973 clone 3 16S ribosomal RNA gene, complete sequence	2649	2649	99%	0.0	99%	JQ924412.1
Streptomyces nitrosporeus strain CGMCC 4.1973 clone 1 16S ribosomal RNA gene, complete sequence	2649	2649	99%	0.0	99%	JQ924411.1
Streptomyces olivoviridis strain CGMCC 4.1739 clone 1 16S ribosomal RNA gene, complete sequence	2647	2647	99%	0.0	99%	JQ924393.1
Streptomyces naraensis gene for 16S rRNA, partial sequence, strain: NBRC 13421	2647	2647	98%	0.0	99%	AB184391.2
Streptomyces fulvorobeus 16S rRNA gene, type strain LMG 19901	2647	2647	97%	0.0	99%	AJ781331.1
Streptomyces parvus gene for 16S rRNA, partial sequence, strain: NBRC 14599	2643	2643	98%	0.0	99%	AB184603.1
Streptomyces setonii gene for 16S rRNA, partial sequence, strain: NBRC 13085	2643	2643	97%	0.0	99%	AB184300.1

Alignments

Streptomyces griseus subsp. griseus NBRC 13350 DNA, complete genome
Sequence ID: **AP009493.1** Length: 8545929 Number of Matches: 6
Range 1: 2102343 to 2103819

Score	Expect	Identities	Gaps	Strand	Frame
2721 bits(1473)	0.0()	1476/1477(99%)	1/1477(0%)	Plus/Plus	

Features:
rRNA-16S ribosomal RNA

```
Query  1        GTTACGACTTCGT-CCAATCGCCAGTCCCACCTTCGACAGCTCCCTCCCACAAGGGGTTG  59
                ||||||||||||| ||||||||||||||||||||||||||||||||||||||||||||||
Sbjct  2102343  GTTACGACTTCGTCCCAATCGCCAGTCCCACCTTCGACAGCTCCCTCCCACAAGGGGTTG  2102402

Query  60       GGCCACCGGCTTCGGGTGTTACCGACTTTCGTGACGTGACGGGCGGTGTGTACAAGGCCC  119
                ||||||||||||||||||||||||||||||||||||||||||||||||||||||||||||
Sbjct  2102403  GGCCACCGGCTTCGGGTGTTACCGACTTTCGTGACGTGACGGGCGGTGTGTACAAGGCCC  2102462

Query  120      GGGAACGTATTCACCGCAGCAATGCTGATCTGCGATTACTAGCAACTCCGACTTCATGGG  179
                ||||||||||||||||||||||||||||||||||||||||||||||||||||||||||||
Sbjct  2102463  GGGAACGTATTCACCGCAGCAATGCTGATCTGCGATTACTAGCAACTCCGACTTCATGGG  2102522

Query  180      GTCGAGTTGCAGACCCCAATCCGAACTGAGACCGGCTTTTTGAGATTCGCTCCGCCTCGC  239
                ||||||||||||||||||||||||||||||||||||||||||||||||||||||||||||
Sbjct  2102523  GTCGAGTTGCAGACCCCAATCCGAACTGAGACCGGCTTTTTGAGATTCGCTCCGCCTCGC  2102582

Query  240      GGCATCGCAGCTCATTGTACCGGCCATTGTAGCACGTGTGCAGCCCAAGACATAAGGGGC  299
                ||||||||||||||||||||||||||||||||||||||||||||||||||||||||||||
Sbjct  2102583  GGCATCGCAGCTCATTGTACCGGCCATTGTAGCACGTGTGCAGCCCAAGACATAAGGGGC  2102642

Query  300      ATGATGACTTGACGTCGTCCCCACCTTCCTCCGAGTTGACCCCGGCAGTCTCCTGTGAGT  359
                ||||||||||||||||||||||||||||||||||||||||||||||||||||||||||||
Sbjct  2102643  ATGATGACTTGACGTCGTCCCCACCTTCCTCCGAGTTGACCCCGGCAGTCTCCTGTGAGT  2102702

Query  360      CCCCATCACCCCGAAGGGCATGCTGGCAACACAGAACAAGGGTTGCGCTCGTTGCGGGAC  419
                ||||||||||||||||||||||||||||||||||||||||||||||||||||||||||||
Sbjct  2102703  CCCCATCACCCCGAAGGGCATGCTGGCAACACAGAACAAGGGTTGCGCTCGTTGCGGGAC  2102762

Query  420      TTAACCCAACATCTCACGACACGAGCTGACGACAGCCATGCACCACCTGTATACCGACCA  479
```

BLAST Results

Questions/comments

Job title: 4_1525

**RID**	TYT8A0E301R (Expires on 09-18 16:25 pm)		
**Query ID**	lcl|Query_130761	**Database Name**	nr
**Description**	4_1525	**Description**	Nucleotide collection (nt)
**Molecule type**	nucleic acid	**Program**	BLASTN 2.8.0+
**Query Length**	1476		

Graphic Summary

Distribution of the top 190 Blast Hits on 100 subject sequences

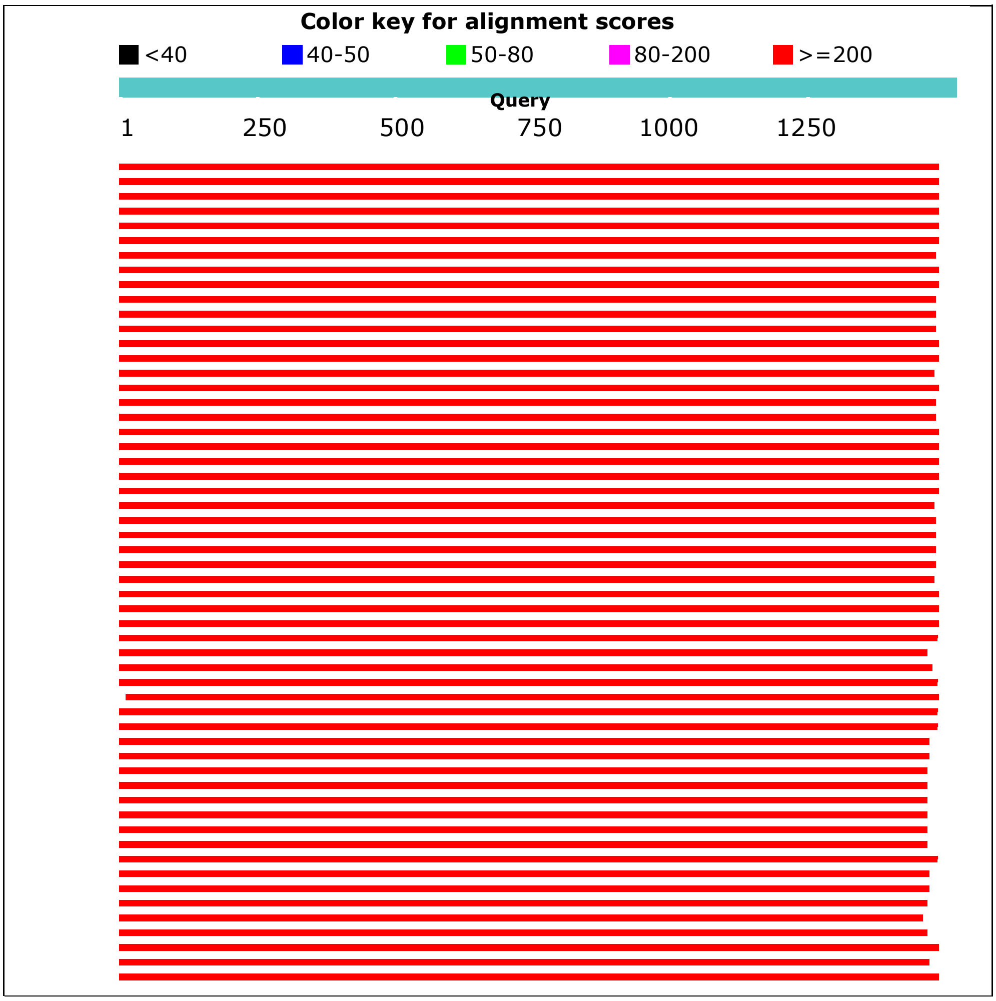

Descriptions

Sequences producing significant alignments:

Description	Max score	Total score	Query cover	E value	Ident	Accession
Streptomyces griseus subsp. griseus NBRC 13350 DNA, complete genome	2721	16327	100%	0.0	99%	AP009493.1
Streptomyces pratensis ATCC 33331, complete genome	2715	16238	100%	0.0	99%	CP002475.1
Streptomyces globisporus strain TFH56 chromosome, complete genome	2710	16238	100%	0.0	99%	CP029361.1
Streptomyces fulvissimus strain DSM 40593 16S ribosomal RNA, partial sequence	2710	2710	100%	0.0	99%	NR_103947.1
Streptomyces fulvissimus DSM 40593, complete genome	2710	16260	100%	0.0	99%	CP005080.1
Streptomyces sp. PAMC26508, complete genome	2710	16257	100%	0.0	99%	CP003990.1
Streptomyces pratensis strain ch24 16S ribosomal RNA, partial sequence	2706	2706	99%	0.0	99%	NR_125621.1
Streptomyces globisporus C-1027, complete genome	2704	16227	100%	0.0	99%	CP013738.1
Streptomyces luridiscabiei strain S63 16S ribosomal RNA, partial sequence	2704	2704	100%	0.0	99%	NR_025155.1
Streptomyces flavolimosus strain CGMCC 2027 16S ribosomal RNA gene, partial sequence	2702	2702	99%	0.0	99%	EF688620.1
Streptomyces praecox strain CGMCC 4.1782 clone 3 16S ribosomal RNA gene, complete sequence	2700	2700	99%	0.0	99%	JQ924404.1
Streptomyces pratensis strain ch24 16S ribosomal RNA, partial sequence	2700	2700	99%	0.0	99%	NR_125619.1
Streptomyces sp. SM18 chromosome, complete genome	2699	16183	100%	0.0	99%	CP029342.1
Streptomyces flavofuscus strain CGMCC 4.1938 clone 3 16S ribosomal RNA gene, complete sequence	2699	2699	99%	0.0	99%	JQ924409.1
Streptomyces griseus strain NRRL B-8030 16S ribosomal RNA gene, partial sequence	2699	2699	99%	0.0	99%	DQ026671.1
Streptomyces sp. Tue6075, complete genome	2695	16157	100%	0.0	99%	CP010833.1
Streptomyces praecox strain CGMCC 4.1782 clone 2 16S ribosomal RNA gene, complete sequence	2695	2695	99%	0.0	99%	JQ924403.1
Streptomyces pratensis strain ch24 16S ribosomal RNA, partial sequence	2695	2695	99%	0.0	99%	NR_125618.1
Streptomyces violaceoruber strain S21, complete genome	2693	16161	100%	0.0	99%	CP020570.1
Streptomyces flavofuscus strain CGMCC 4.1938 clone 2 16S ribosomal RNA gene, complete sequence	2693	2693	99%	0.0	99%	JQ924408.1
Streptomyces flavofuscus strain CGMCC 4.1938 clone 1 16S ribosomal RNA gene, complete sequence	2693	2693	99%	0.0	99%	JQ924407.1
Streptomyces sp. SirexAA-E, complete genome	2693	16133	100%	0.0	99%	CP002993.1
Streptomyces flavofuscus strain NRRL B-2594 16S ribosomal RNA gene, partial sequence	2691	2691	100%	0.0	99%	EF178690.1

Description	Max score	Total score	Query cover	E value	Ident	Accession
Streptomyces microflavus strain NRRL B-2156 16S ribosomal RNA gene, partial sequence	2691	2691	99%	0.0	99%	DQ445795.1
Streptomyces praecox strain CGMCC 4.1782 clone 5 16S ribosomal RNA gene, complete sequence	2689	2689	99%	0.0	99%	JQ924406.1
Streptomyces praecox strain CGMCC 4.1782 clone 4 16S ribosomal RNA gene, complete sequence	2689	2689	99%	0.0	99%	JQ924405.1
Streptomyces praecox strain CGMCC 4.1782 clone 1 16S ribosomal RNA gene, complete sequence	2689	2689	99%	0.0	99%	JQ924402.1
Streptomyces pratensis strain ch24 16S ribosomal RNA, partial sequence	2689	2689	99%	0.0	99%	NR_125620.1
Streptomyces anulatus strain NRRL B-2000 16S ribosomal RNA gene, partial sequence	2689	2689	99%	0.0	99%	DQ026637.1
Streptomyces flavogriseus partial 16S rRNA gene, type strain CBS 101.34T	2687	2687	100%	0.0	99%	AJ494864.1
Streptomyces baarnensis strain NRRL B-1902 16S ribosomal RNA gene, partial sequence	2686	2686	100%	0.0	99%	EF178688.1
Streptomyces sp. CFMR 7 strain CFMR-7, complete genome	2682	15924	100%	0.0	99%	CP011522.1
Streptomyces flavovirens strain CGMCC 4.575 clone 1 16S ribosomal RNA gene, complete sequence	2682	2682	99%	0.0	99%	JQ924386.1
Streptomyces griseus subsp. griseus gene for 16S rRNA, partial sequence, strain: NBRC 15744	2680	2680	98%	0.0	99%	AB184699.1
Streptomyces argenteolus strain JCM 4623 16S ribosomal RNA, partial sequence	2680	2680	99%	0.0	99%	NR_112120.1
Streptomyces flavovirens strain CGMCC 4.575 clone 2 16S ribosomal RNA gene, complete sequence	2678	2678	99%	0.0	99%	JQ924387.1
Streptomyces sp. YM5-799 gene for 16S rRNA, partial sequence	2678	2678	99%	0.0	99%	AB534176.1
Streptomyces flavovirens strain CGMCC 4.575 clone 5 16S ribosomal RNA gene, complete sequence	2676	2676	99%	0.0	99%	JQ924389.1
Streptomyces flavovirens strain CGMCC 4.575 clone 4 16S ribosomal RNA gene, complete sequence	2676	2676	99%	0.0	99%	JQ924388.1
Streptomyces microflavus strain NRRL B-1332 16S ribosomal RNA gene, partial sequence	2675	2675	98%	0.0	99%	EF178673.1
Streptomyces paresii 16S rRNA gene, type strain LMG 23704T	2675	2675	98%	0.0	99%	AJ969177.1
Streptomyces anulatus gene for 16S rRNA, partial sequence, strain: NBRC 13369	2673	2673	98%	0.0	99%	AB184875.1
Streptomyces cavourensis subsp. washingtonensis gene for 16S rRNA, partial sequence, strain: NBRC 15391	2673	2673	98%	0.0	99%	AB184642.1
Streptomyces acidoresistans gene for 16S rRNA, partial sequence, strain: NBRC 13442	2673	2673	98%	0.0	99%	AB184406.1
Streptomyces praecox gene for 16S rRNA, partial sequence, strain: NBRC 13073	2673	2673	98%	0.0	99%	AB184293.1
Streptomyces alboviridis gene for 16S rRNA, partial sequence, strain: NBRC 13013	2673	2673	98%	0.0	99%	AB184256.1

Description	Max score	Total score	Query cover	E value	Ident	Accession
Streptomyces argenteolus gene for 16S rRNA, partial sequence, strain: NBRC 12841	2673	2673	98%	0.0	99%	AB184187.1
Streptomyces finlayi strain CGMCC 4.1436 clone 1 16S ribosomal RNA gene, complete sequence	2669	2669	99%	0.0	99%	JQ924390.1
Streptomyces rubiginosohelvolus gene for 16S rRNA, partial sequence, strain: NBRC 12912	2669	2669	98%	0.0	99%	AB184240.2
Streptomyces microflavus gene for 16S rRNA, partial sequence, strain: NBRC 13062	2669	2669	98%	0.0	99%	AB184284.1
Streptomyces fulvorobeus gene for 16S rRNA, partial sequence, strain: NBRC 15897	2667	2667	98%	0.0	99%	AB184711.1
Streptomyces griseus subsp. griseus gene for 16S rRNA, partial sequence, strain: NBRC 12875	2667	2667	98%	0.0	99%	AB184211.1
Streptomyces griseinus gene for 16S rRNA, partial sequence, strain: NBRC 12869	2667	2667	98%	0.0	99%	AB184205.1
Streptomyces sp. S8, complete genome	2665	15950	100%	0.0	99%	CP015362.1
Streptomyces flavofuscus gene for 16S rRNA, partial sequence, strain: NBRC 100768	2665	2665	98%	0.0	99%	AB249935.1
Streptomyces clavifer strain NRRL B-2557 16S ribosomal RNA gene, partial sequence	2663	2663	99%	0.0	99%	DQ026670.1
Streptomyces albovinaceus gene for 16S rRNA, partial sequence, strain: NBRC 12739	2663	2663	98%	0.0	99%	AB249958.1
Streptomyces erumpens gene for 16S rRNA, partial sequence, strain: NBRC 15403	2663	2663	97%	0.0	99%	AB184654.1
Streptomyces baarnensis gene for 16S rRNA, partial sequence, strain: NBRC 14727	2663	2663	98%	0.0	99%	AB184615.1
Streptomyces fimicarius gene for 16S rRNA, partial sequence, strain: NBRC 13037	2663	2663	98%	0.0	99%	AB184269.1
Streptomyces globisporus subsp. globisporus gene for 16S rRNA, partial sequence, strain: NBRC 12867	2663	2663	98%	0.0	99%	AB184203.1
Streptomyces cavourensis strain TJ430 chromosome, complete genome	2662	15856	100%	0.0	99%	CP030930.1
Streptomyces bacillaris strain ATCC 15855 chromosome, complete genome	2662	15972	100%	0.0	99%	CP029378.1
Streptomyces cavourensis strain 1AS2a chromosome, complete genome	2662	15928	100%	0.0	99%	CP024957.1
Streptomyces globisporus subsp. globisporus strain NRRL B-2872 16S ribosomal RNA gene, partial sequence	2662	2662	98%	0.0	99%	EF178686.1
Streptomyces griseolus gene for 16S rRNA, partial sequence, strain: NBRC 3415	2662	2662	98%	0.0	99%	AB184768.1
Streptomyces puniceus gene for 16S rRNA, partial sequence, strain: NBRC 12811	2662	2662	98%	0.0	99%	AB184163.1
Streptomyces halstedii gene for 16S rRNA, partial sequence, strain: NBRC 12783	2662	2662	98%	0.0	99%	AB184142.1

Description	Max score	Total score	Query cover	E value	Ident	Accession
Streptomyces atratus strain SCSIO_ZH16 chromosome, complete genome	2660	15961	100%	0.0	99%	CP027306.1
Streptomyces flavovirens strain NRRL B-2685 16S ribosomal RNA gene, partial sequence	2660	2660	99%	0.0	99%	DQ026635.1
Streptomyces pluricolorescens gene for 16S rRNA, partial sequence, strain: NBRC 12808	2660	2660	98%	0.0	99%	AB184162.1
Streptomyces olivoviridis strain CGMCC 4.1739 clone 2 16S ribosomal RNA gene, complete sequence	2658	2658	99%	0.0	99%	JQ924394.1
Streptomyces finlayi strain CGMCC 4.1436 clone 2 16S ribosomal RNA gene, complete sequence	2658	2658	99%	0.0	99%	JQ924391.1
Streptomyces californicus gene for 16S rRNA, partial sequence, strain: NBRC 12750	2658	2658	98%	0.0	99%	AB184116.2
Streptomyces cinereorectus gene for 16S rRNA, partial sequence, strain: NBRC 15395	2658	2658	98%	0.0	99%	AB184646.1
Streptomyces mutomycini strain CGMCC 4.1747 clone 3 16S ribosomal RNA gene, complete sequence	2656	2656	99%	0.0	99%	JQ924399.1
Streptomyces halstedii strain NRRL B-1238 16S ribosomal RNA gene, partial sequence	2656	2656	98%	0.0	99%	EF178695.1
Streptomyces parvus gene for 16S rRNA, partial sequence, strain: NBRC 3388	2656	2656	98%	0.0	99%	AB184756.1
Streptomyces californicus gene for 16S rRNA, partial sequence, strain: NBRC 3386	2656	2656	98%	0.0	99%	AB184755.1
Streptomyces badius gene for 16S rRNA, partial sequence, strain: NBRC 12745	2656	2656	98%	0.0	99%	AB184114.1
Streptomyces lunaelactis strain MM109 chromosome, complete genome	2654	15660	100%	0.0	99%	CP026304.1
Streptomyces nitrosporeus strain CGMCC 4.1973 clone 4 16S ribosomal RNA gene, complete sequence	2654	2654	99%	0.0	99%	JQ924413.1
Streptomyces anulatus strain NRRL B-2873 16S ribosomal RNA gene, partial sequence	2654	2654	97%	0.0	99%	DQ026639.1
Streptomyces sindenensis gene for 16S rRNA, partial sequence, strain: NBRC 3399	2654	2654	98%	0.0	99%	AB184759.1
Streptomyces mediolani gene for 16S rRNA, partial sequence, strain: NBRC 15427	2654	2654	98%	0.0	99%	AB184674.1
Streptomyces olivoviridis strain CGMCC 4.1739 clone 5 16S ribosomal RNA gene, complete sequence	2652	2652	99%	0.0	99%	JQ924396.1
Streptomyces olivoviridis strain CGMCC 4.1739 clone 4 16S ribosomal RNA gene, complete sequence	2652	2652	99%	0.0	99%	JQ924395.1
Kitasatospora albolonga strain YIM 101047, complete genome	2651	15858	100%	0.0	99%	CP020563.1
Streptomyces mutomycini strain CGMCC 4.1747 clone 1 16S ribosomal RNA gene, complete sequence	2651	2651	99%	0.0	99%	JQ924397.1

Description	Max score	Total score	Query cover	E value	Ident	Accession
Streptomyces flavovirens gene for 16S rRNA, partial sequence, strain: NBRC 3197	2651	2651	98%	0.0	99%	AB184827.1
Streptomyces ornatus gene for 16S rRNA, partial sequence, strain: NBRC 13069	2651	2651	97%	0.0	99%	AB184290.1
Streptomyces flavogriseus gene for 16S rRNA, partial sequence, strain: NBRC 13040	2651	2651	98%	0.0	99%	AB184271.1
Streptomyces flavovirens gene for 16S rRNA, partial sequence, strain: NBRC 12771	2651	2651	98%	0.0	99%	AB184133.1
Streptomyces nitrosporeus strain CGMCC 4.1973 clone 3 16S ribosomal RNA gene, complete sequence	2649	2649	99%	0.0	99%	JQ924412.1
Streptomyces nitrosporeus strain CGMCC 4.1973 clone 1 16S ribosomal RNA gene, complete sequence	2649	2649	99%	0.0	99%	JQ924411.1
Streptomyces olivoviridis strain CGMCC 4.1739 clone 1 16S ribosomal RNA gene, complete sequence	2647	2647	99%	0.0	99%	JQ924393.1
Streptomyces naraensis gene for 16S rRNA, partial sequence, strain: NBRC 13421	2647	2647	98%	0.0	99%	AB184391.2
Streptomyces fulvorobeus 16S rRNA gene, type strain LMG 19901	2647	2647	97%	0.0	99%	AJ781331.1
Streptomyces parvus gene for 16S rRNA, partial sequence, strain: NBRC 14599	2643	2643	98%	0.0	99%	AB184603.1
Streptomyces setonii gene for 16S rRNA, partial sequence, strain: NBRC 13085	2643	2643	97%	0.0	99%	AB184300.1

Alignments

Streptomyces griseus subsp. griseus NBRC 13350 DNA, complete genome
Sequence ID: **AP009493.1** Length: 8545929 Number of Matches: 6
Range 1: 2102343 to 2103819

Score	Expect	Identities	Gaps	Strand	Frame
2721 bits(1473)	0.0()	1476/1477(99%)	1/1477(0%)	Plus/Plus	

Features:
rRNA-16S ribosomal RNA

```
Query  1        GTTACGACTTCGT-CCAATCGCCAGTCCCACCTTCGACAGCTCCCTCCCACAAGGGGTTG  59
                ||||||||||||| ||||||||||||||||||||||||||||||||||||||||||||||
Sbjct  2102343  GTTACGACTTCGTCCCAATCGCCAGTCCCACCTTCGACAGCTCCCTCCCACAAGGGGTTG  2102402

Query  60       GGCCACCGGCTTCGGGTGTTACCGACTTTCGTGACGTGACGGGCGGTGTGTACAAGGCCC  119
                ||||||||||||||||||||||||||||||||||||||||||||||||||||||||||||
Sbjct  2102403  GGCCACCGGCTTCGGGTGTTACCGACTTTCGTGACGTGACGGGCGGTGTGTACAAGGCCC  2102462

Query  120      GGGAACGTATTCACCGCAGCAATGCTGATCTGCGATTACTAGCAACTCCGACTTCATGGG  179
                ||||||||||||||||||||||||||||||||||||||||||||||||||||||||||||
Sbjct  2102463  GGGAACGTATTCACCGCAGCAATGCTGATCTGCGATTACTAGCAACTCCGACTTCATGGG  2102522

Query  180      GTCGAGTTGCAGACCCCAATCCGAACTGAGACCGGCTTTTTGAGATTCGCTCCGCCTCGC  239
                ||||||||||||||||||||||||||||||||||||||||||||||||||||||||||||
Sbjct  2102523  GTCGAGTTGCAGACCCCAATCCGAACTGAGACCGGCTTTTTGAGATTCGCTCCGCCTCGC  2102582

Query  240      GGCATCGCAGCTCATTGTACCGGCCATTGTAGCACGTGTGCAGCCCAAGACATAAGGGGC  299
                ||||||||||||||||||||||||||||||||||||||||||||||||||||||||||||
Sbjct  2102583  GGCATCGCAGCTCATTGTACCGGCCATTGTAGCACGTGTGCAGCCCAAGACATAAGGGGC  2102642

Query  300      ATGATGACTTGACGTCGTCCCCACCTTCCTCCGAGTTGACCCCGGCAGTCTCCTGTGAGT  359
                ||||||||||||||||||||||||||||||||||||||||||||||||||||||||||||
Sbjct  2102643  ATGATGACTTGACGTCGTCCCCACCTTCCTCCGAGTTGACCCCGGCAGTCTCCTGTGAGT  2102702

Query  360      CCCCATCACCCCGAAGGGCATGCTGGCAACACAGAACAAGGGTTGCGCTCGTTGCGGGAC  419
                ||||||||||||||||||||||||||||||||||||||||||||||||||||||||||||
Sbjct  2102703  CCCCATCACCCCGAAGGGCATGCTGGCAACACAGAACAAGGGTTGCGCTCGTTGCGGGAC  2102762

Query  420      TTAACCCAACATCTCACGACACGAGCTGACGACAGCCATGCACCACCTGTATACCGACCA  479
```

Antibiotic test

Antibiotic test	
**EI:**	*Streptomyces* sp. SN25_8.1
**SG:**	*Streptomyces griseus* subsp. griseus DSM 40236^T
**ST:**	Streptomycin

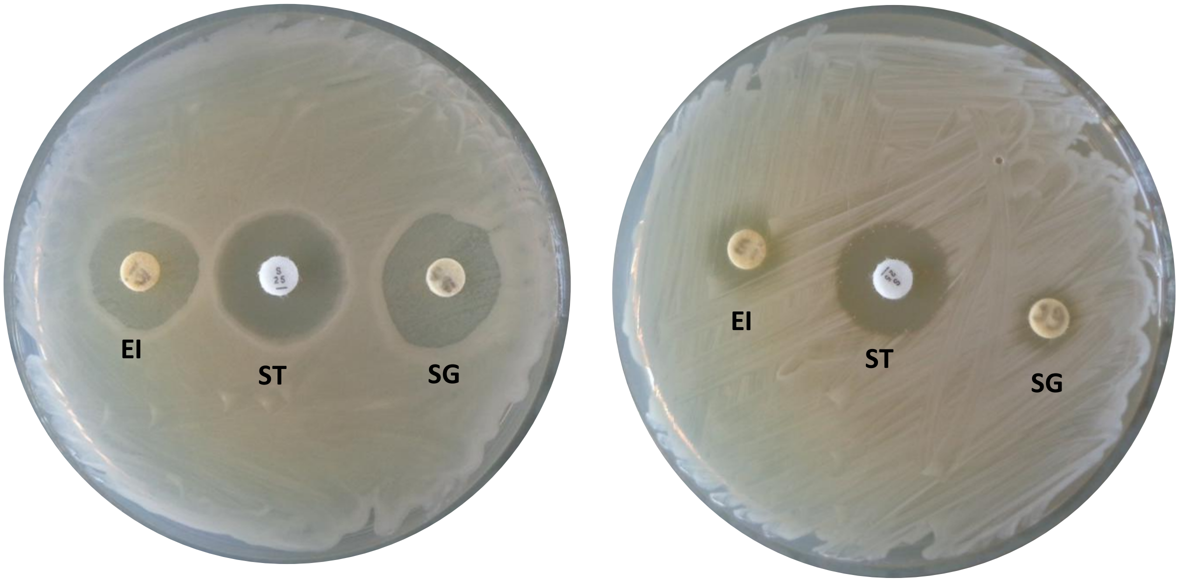

<u>Antibiotic test</u> Left plate: *E. coli* DSM 498, Right Plate: *S. lentus* DSM 20352

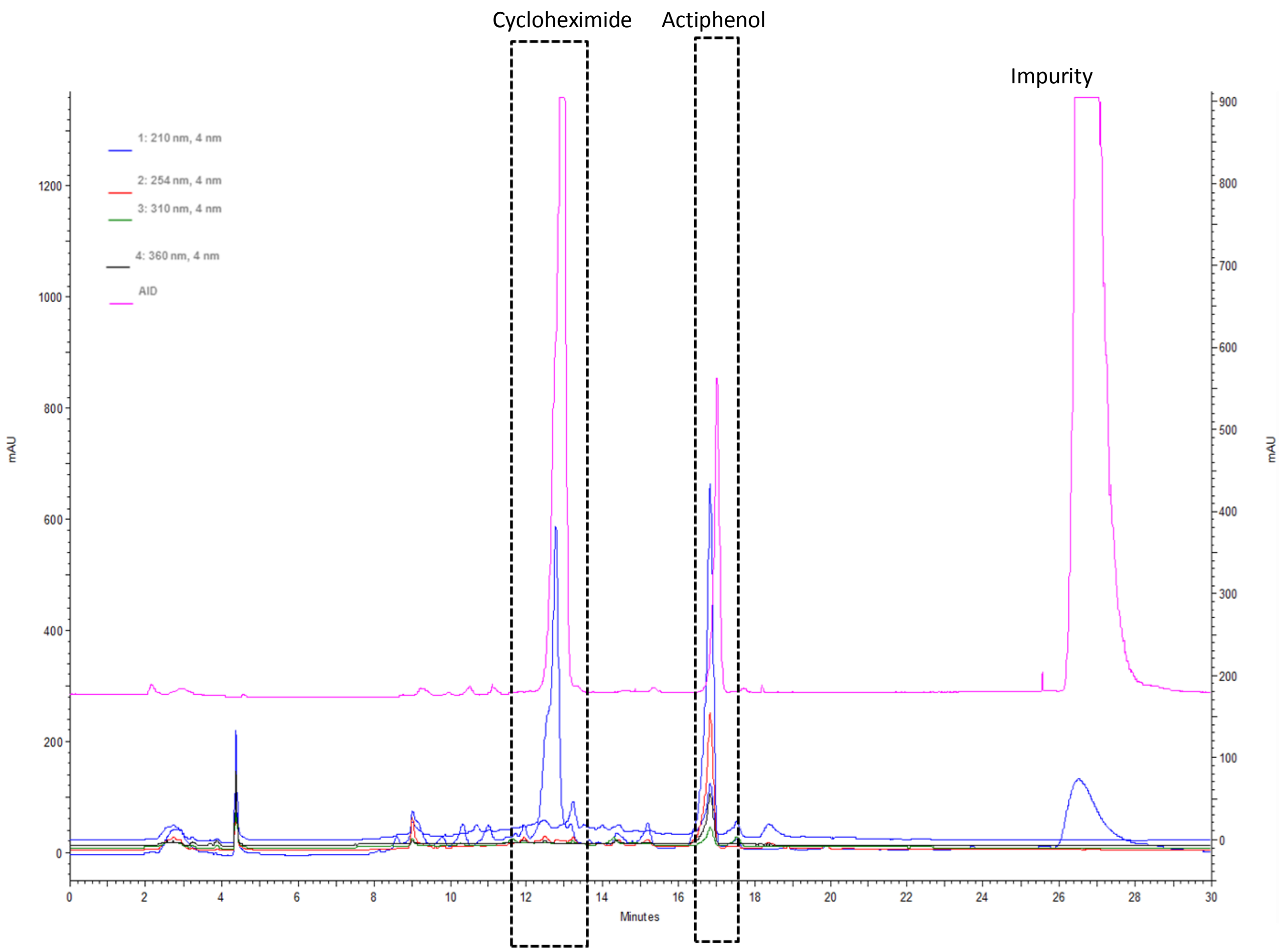

HPLC –ELSD profile *of Streptomyces* sp. SN25_8.1.

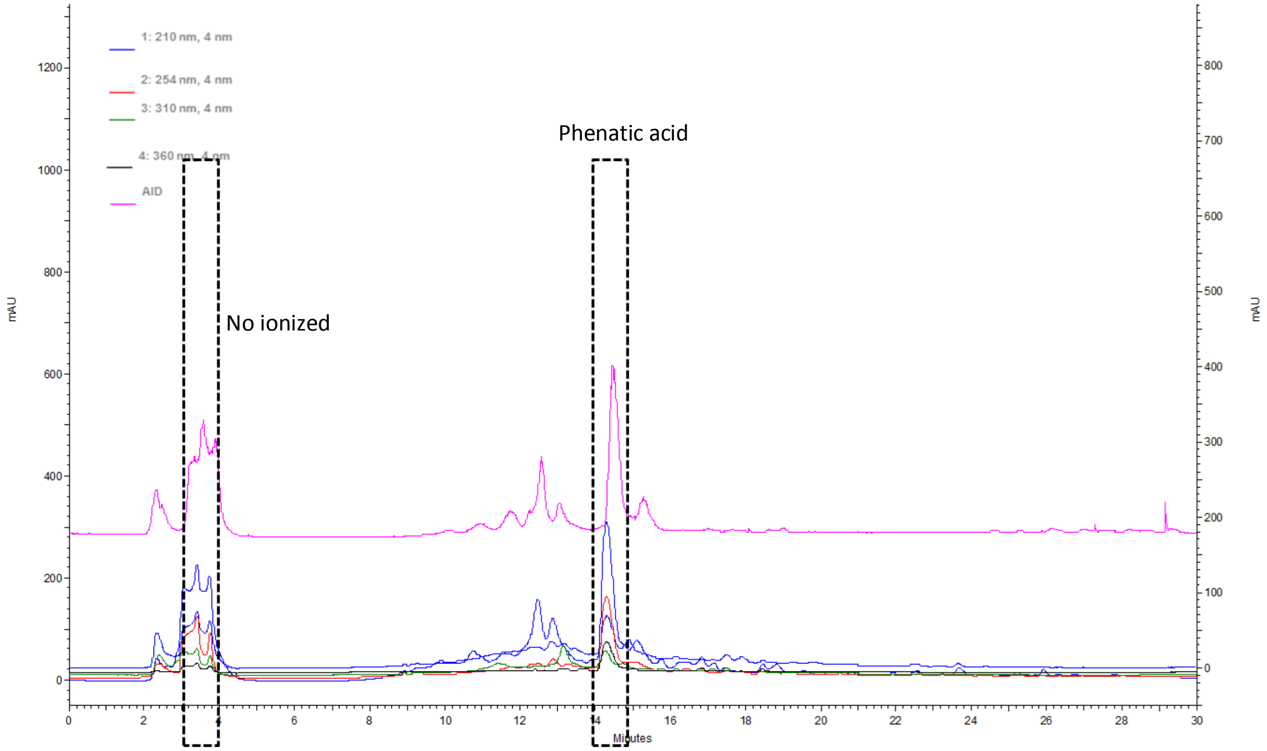

HPLC –ELSD profile *of Streptomyces griseus* subsp. griseus DSM 40236ᵀ.
